# POCUS for acute abdominal pain: practical scan protocols on gastrointestinal diseases and an evidence review

**DOI:** 10.1007/s40477-025-01088-7

**Published:** 2025-09-29

**Authors:** Andrea Boccatonda, Alice Brighenti, Valeria Tiraferri, Marisol Doglioli, Luca Iazzetta, Lucia De Meis, Ehsan Safai Zadeh, Christoph Frank Dietrich, Carla Serra

**Affiliations:** 1 Diagnostic and Therapeutic Interventional Ultrasound Unit, IRCCS Azienda Ospedaliero-Universitaria di Bologna, Policlinico Sant’Orsola-Malpighi, via Massarenti n 9, 40138 Bologna, Italy; 2 IRCCS San Raffaele Hospital, Vita-Salute San Raffaele University, Milan, Italy; 3 Division of Gynaecology and Human Reproduction Physiopathology, IRCCS Azienda Ospedaliero-Universitaria di Bologna, Bologna, Italy; 4 Department of Medical and Surgical Sciences, DIMEC, University of Bologna, Bologna, Italy; 5 Department of Biomedical Imaging and Image-Guided Therapy, Medical University of Vienna, 1019 Vienna, Austria; 6 Department Allgemeine Innere Medizin (DAIM), Kliniken Hirslanden Bern, Beau Site, Salem und Permanence, 3013 Bern, Switzerland

**Keywords:** Ultrasound, Pain, Abdomen, POCUS, Pelvis

## Abstract

Acute abdominal pain is a frequent emergency department presentation requiring prompt and accurate diagnosis to guide timely management. Ultrasound imaging plays a critical role in the differential diagnosis of this symptom, offering several advantages including wide availability, cost-effectiveness, and real-time assessment without ionizing radiation. Color and spectral Doppler further enhance diagnostic accuracy by allowing the assessment of blood flow and vascular patterns, which is crucial for identifying ischemic processes. Additionally, ultrasound can help distinguish between gynecological and non-gynecological conditions, such as appendicitis, urinary tract pathologies, or gastrointestinal abnormalities, thereby guiding more targeted investigative pathways or treatment modalities. The rapid, bedside application of ultrasound is especially valuable in unstable patients, ensuring expedited triage and intervention. In certain cases, a negative or inconclusive ultrasound may necessitate further imaging with computed tomography or magnetic resonance imaging. However, ultrasound remains the first-line modality, particularly in pregnant women, to minimize radiation exposure. By integrating ultrasound findings with clinical data and laboratory results, clinicians can establish a precise diagnosis, avoid unnecessary procedures, and initiate timely therapeutic interventions, ultimately improving patient outcomes.

## Introduction

Acute abdominal pain (ABP) is a common reason for emergency department visits, affecting a broad spectrum of the population, from young individuals to the elderly [1]. The differential diagnosis of ABP is extensive and includes a variety of conditions such as gastrointestinal (GI), urinary, vascular and gynecological diseases [2–5]. Accurate diagnosis is challenging due to the close anatomical and physiological relationships between pelvic structures, the overlap of symptoms, and the similar presentation of various pelvic pathologies [6, 7]. Ultrasound (US) (both transvaginal and transabdominal) is a safe, low-cost, widely available diagnostic method without ionizing radiation, which can be quickly utilized in an emergency setting for evaluating patients with ABP [8]. This technique also offers the advantage of relatively high sensitivity and specificity for detecting pelvic pathology [1]. These capabilities make US an attractive first-line test for many causes of pelvic pain, reserving computed tomography (CT) or magnetic resonance (MRI) for equivocal cases, suspected complications, or pre-operative planning. At the same time, performance is operator- and patient-dependent (e.g., body habitus, pain tolerance), and evidence is uneven across conditions.

Point-of-care US (POCUS) is the acquisition and real-time interpretation of US images by the treating clinician at the bedside [9], integrated with the history and physical exam to answer focused clinical questions or guide procedures. It is problem-oriented (rule-in/rule-out), repeatable, and radiation-free, and is intended to complement—not replace—comprehensive radiology-performed US or cross-sectional imaging [10–12].

In addition to conventional greyscale and color-Doppler [13], contrast-enhanced ultrasound (CEUS) can further increase diagnostic confidence by depicting microvascular perfusion in real time [12, 14]. Because CEUS is rapid, radiation-free, and often more accessible than CT or MRI in resource-limited or remote institutions, it represents a practical adjunct when cross-sectional imaging is not immediately available or when bedside decision-making is required [12, 15].

Our review aims to knit together what clinicians need to see with what the literature shows. We illustrate standardized scanning approaches and hallmark sonographic signs for the major GI etiologies of acute pelvic pain, and we embed each image-based teaching point within a concise synthesis of contemporary evidence on POCUS examination.

To support these aims, we conducted a targeted literature search. We searched PubMed/MEDLINE, Embase, Scopus, Web of Science, Cochrane CENTRAL, and Google Scholar for studies published from January 1, 2019, through August 31, 2025. We included English language studies on POCUS enrolling adults and pediatric patients with acute abdominal or abdominopelvic pain with specific GI conditions. We restricted results to the following publication types (where supported by each database): Clinical Study; Clinical Trial; Clinical Trial Protocol; Comparative Study; Controlled Clinical Trial; Meta-Analysis; Multicenter Study; Network Meta-Analysis; Observational Study; Randomized Controlled Trial; Systematic Review; Validation Study.

## Appendicitis

**Fig. 1:**
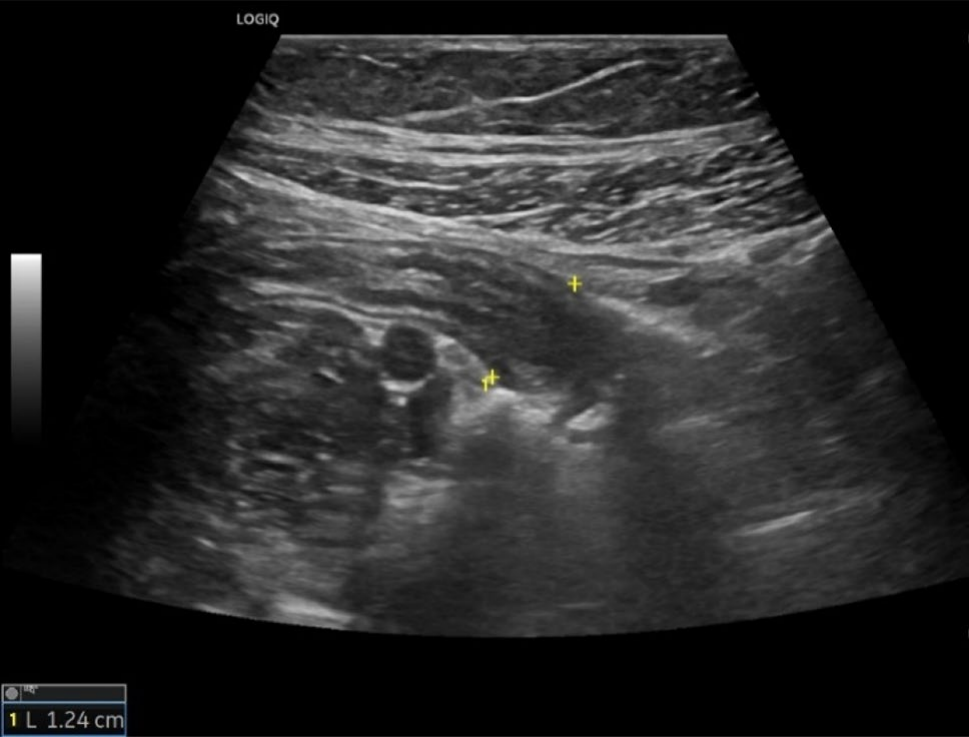
The appendix appears thickened with a maximum diameter of 12 mm with a pronounced fat tissue stranding, and the wall stratification is preserved, consistent with findings of acute appendicitis

**Fig. 2:**
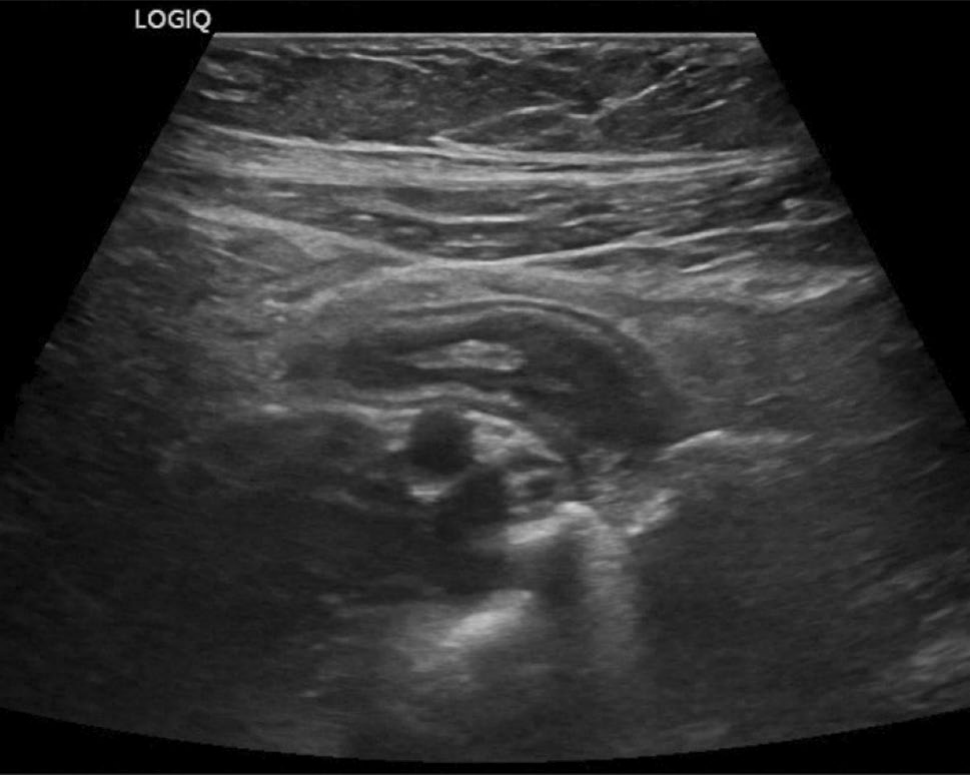
The appendix appears thickened with a maximum diameter of 12 mm with a pronounced fat tissue stranding, and the wall stratification is preserved, consistent with findings of acute appendicitis

There are two main methods to evaluate an inflamed appendix. A systematic approach can be used, which involves identifying the cecum and terminal ileum, and locating the origin of the appendix approximately 2–3 cm from the medial margin of the cecum [16]. Alternatively, the appendix can be searched for at the point of maximum tenderness. Direct signs of appendicitis are maximal tenderness over the thickened appendix and incompressibility of the inflamed appendix [17] during the ultrasound examination [18], a maximum outer diameter greater than 6 mm [19], hypervascularity on color-Doppler in uncomplicated cases [20], and the presence of appendicoliths [21] (Figs. 1 and 2). Indirect ultrasound signs of appendicitis are related to surrounding tissue inflammation and include peri-appendiceal fluid, mesenteric lymphadenopathy, and hyperechoic peri-appendiceal tissue, indicative of mesenteric fat hypertrophy [18]. A relevant technical limitation is the retrocecal appendix, one of the most frequent anatomic position, which can be difficult to visualize with graded compression and is a recognized cause of false-negative studies [22]. When appendiceal visualization is suboptimal, we recommend systematic cecal and terminal ileal tracking, targeted scanning at the point of maximal tenderness, patient repositioning, and a low threshold for confirmatory imaging when clinical suspicion remains high [23].

In cases of complications, distinctive signs include abscesses, extraluminal air, intraluminal air, extraluminal appendicolith, intraluminal appendicolith, peri-appendiceal fat stranding, peri-appendiceal fluid, ileus, and ascites [24].

## Systematic review of recent evidence

All four imaging modalities—US, POCUS, CT, and MRI—show high diagnostic accuracy for pediatric acute appendicitis (Table 1). In a 37-study pediatric meta-analysis (22 US, 4 POCUS, 4 CT, 13 MRI; ~ 25,000 patients total), pooled sensitivity/specificity were: US 0.93/0.89; POCUS 0.80/0.93; CT 0.96/0.98; MRI 0.96/0.98. Diagnostic odds ratios were highest for CT (~ 864) and MRI (~ 1,030), lower for US (~ 115) and POCUS (~ 54), with substantial heterogeneity for US/POCUS. Overall modality differences were not statistically significant (*P* = 0.07) [25].

**Table 1:** Summary of included studies on POCUS for acute appendicitis

Study	Year	Population/setting	*N* (studies/patients)	sensitivity (95% CI)	Specificity (95% CI)	Other performance	Notes
Castro-luna et al. [25]	2025	Children & adolescents (< 21 y), multi-study SR/MA	4/280	0.80 (0.61–0.91)	0.93 (0.83–0.98)	DOR 53.97	Higher heterogeneity vs CT/MRI
Dessie et al. [26]	2025	Pediatric ED (ages 5–17), 9 sites; POCUS severity staging	Prospective single study/72	Complicated: 1.00 (0.77–1.00)	Complicated: 0.65 (0.52–0.78)	Mean scan time ~ 8 min; Ease-of-use 94%; Acceptance 100%	Accurately ruled out uncomplicated cases; adjunct for management
Cho & Oh [27]	2023	ED, bedside US (21 studies)	SR/MA/21 studies	0.81 (0.78–0.83)	0.87 (0.85–0.88)	AUC 0.925; *I*^2^: Sens 91.7%, Spec 90.9%	High heterogeneity across included studies
Becker et al. [66]	2022	Adult ED; POCUS by EPs	Prospective multicenter/256	0.85 (0.74–0.92)	0.63 (0.56–0.70)	LR + 2.29; LR − 0.24; Prevalence 28.1%	Moderate accuracy; not definitive alone in undifferentiated ED population
Scheier et al	2024	Pediatric ED; 8-year retrospective review of POCUS for appendicitis	Single-center/999 (POCUS subset: 360 with appendicitis; 19 without)	NR	NR	POCUS use and correctness increased; ED LOS ↓ ~ 20 min; opioid administration ↑	Highest accuracy ages 5–10; lowest in females 10–15; strong rule-in among confirmed cases; limited rule-out in non-appendicitis (6/19 correctly negative)

ED Emergency Department, POCUS Point-of-Care Ultrasound, SR/MA Systematic Review/Meta-analysis, CI Confidence Interval, DOR Diagnostic Odds Ratio, LR Likelihood Ratio; LOS Length of Stay.

Complementary evidence from emergency department settings indicates that POCUS can aid triage and management: a pediatric multicenter feasibility study using POCUS with Puylaert submucosal staging accurately ruled out complicated appendicitis (sensitivity 100% [95% CI 77–100%], specificity 65% [52–78%]); physicians found it quick (≈8 min) and well accepted by families [26]. Broader ED meta-analytic data (mixed populations) also supports ultrasound’s high overall accuracy (AUC ~ 0.92) but underscores marked heterogeneity (*I*^2^ ≈ 91–91%) [27].

In a retrospective cohort of children diagnosed with appendicitis or periappendiceal abscess from June 2016–June 2024, investigators examined POCUS use, performance, and operational impact in the pediatric ED [28]. Among 999 children, 845 had histology-confirmed appendicitis and 69 did not (histology negative) [28]. POCUS was performed in 360/845 (43%) confirmed cases and 19/69 (28%) non-appendicitis cases. Over time, both the volume of POCUS examinations and the proportion correctly identifying appendicitis increased. Diagnostic performance varied by age/sex, with highest accuracy in ages 5–10 years and lowest in females 10–15 years [28]. POCUS showed strong rule-in performance in confirmed appendicitis (reported correctness in 96% of positive POCUS among cases with pathologic appendicitis), but limited rule-out capability among non-appendicitis patients (6/19 POCUS correctly negative) [28]. Clinically, POCUS use was associated with greater opioid administration and a mean 20-min reduction in pediatric ED length of stay [28].

In adults, a large multicenter ED study reported moderate POCUS performance (sensitivity 0.85, specificity 0.63), suggesting POCUS alone is insufficient as a definitive test in undifferentiated populations and should be integrated with clinical assessment and/or confirmatory imaging [27].

## Diverticulitis

**Fig. 3:**
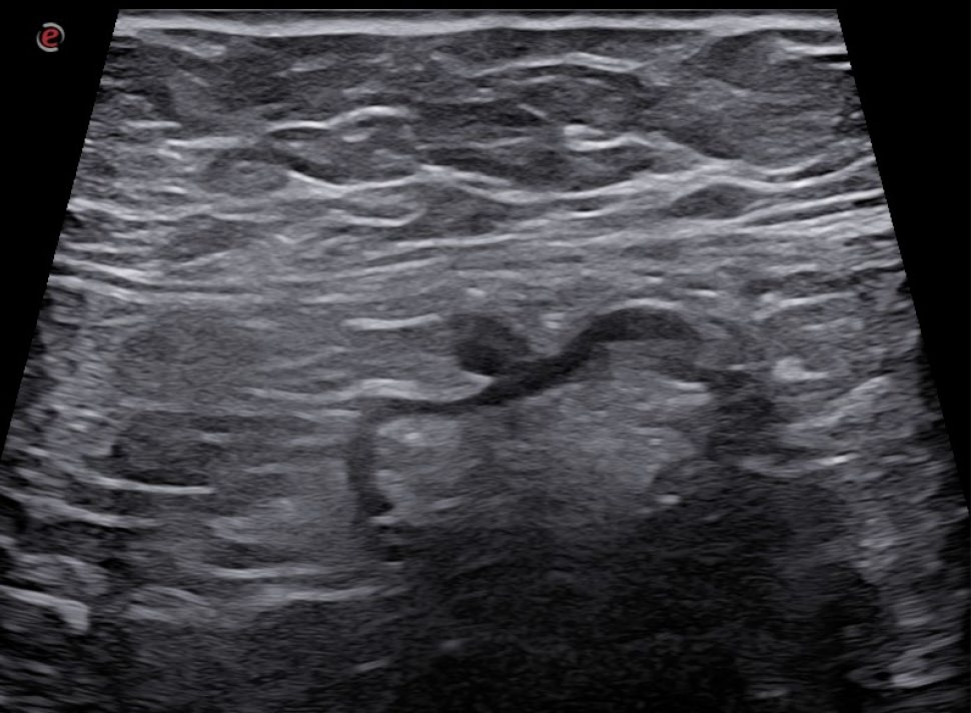
At the level of the sigmoid colon, several wall diverticula are visible, consistent with diverticulosis. The organ wall shows areas with thickness at the upper limits of normal (2–3 mm). Furthermore, fat stranding is visible around the inflammatory area

**Fig. 4:**
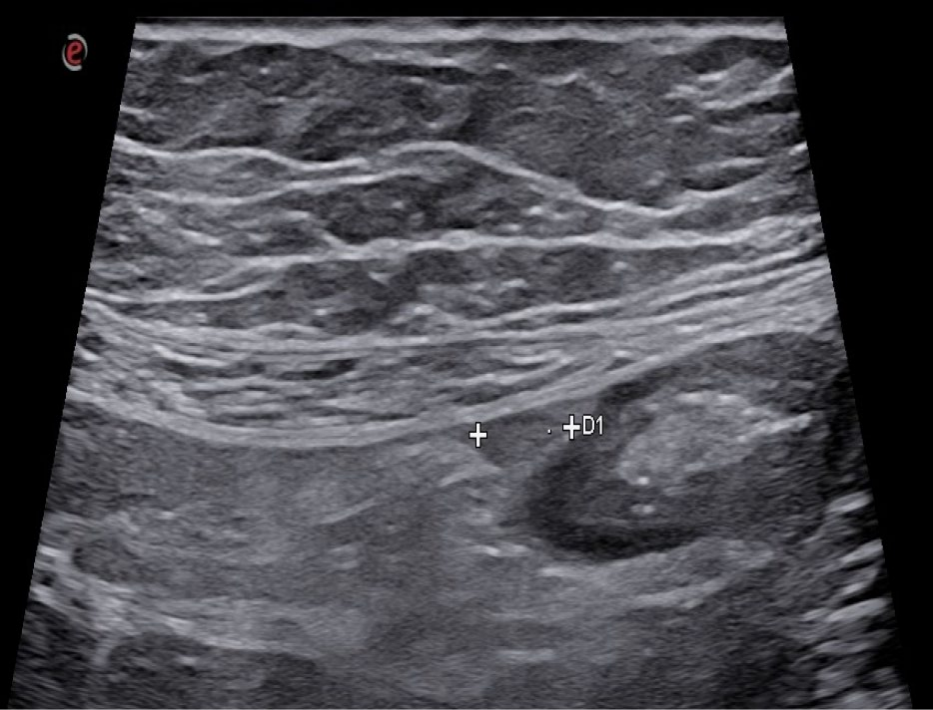
At the level of the sigmoid colon, several wall diverticula are visible, consistent with diverticulosis. The organ wall shows areas with thickness at the upper limits of normal (2–3 mm). Furthermore, fat stranding is visible around the inflammatory area

**Fig. 5:**
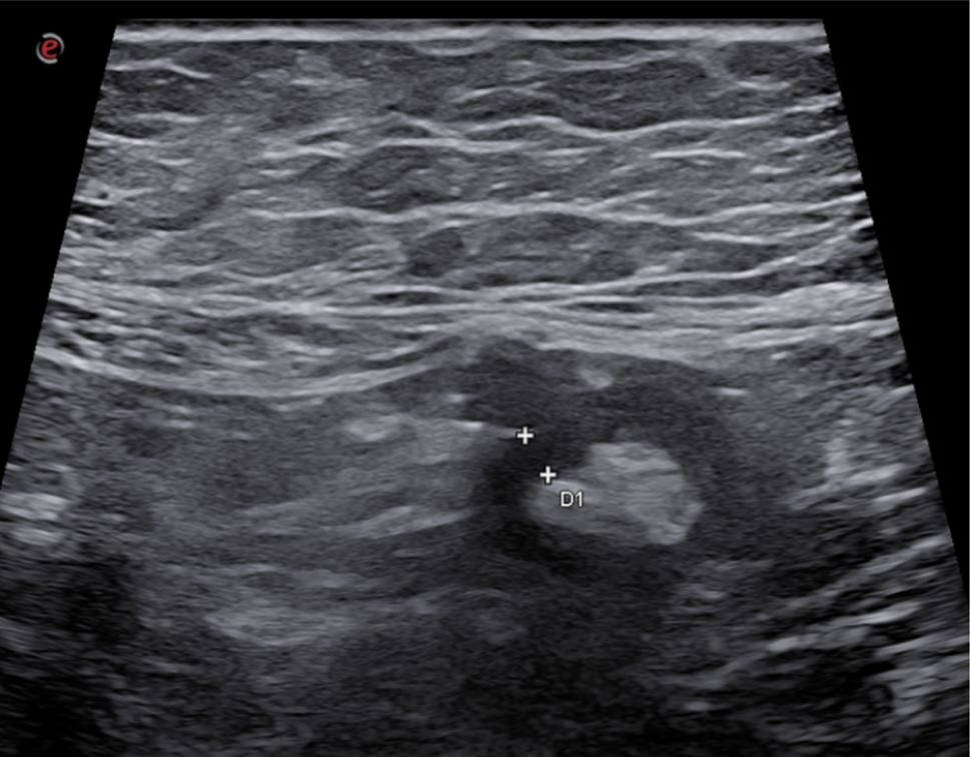
At the level of the sigmoid colon, several wall diverticula are visible, consistent with diverticulosis. The organ wall shows areas with thickness at the upper limits of normal (2–3 mm). Furthermore, fat stranding is visible around the inflammatory area

**Fig. 6:**
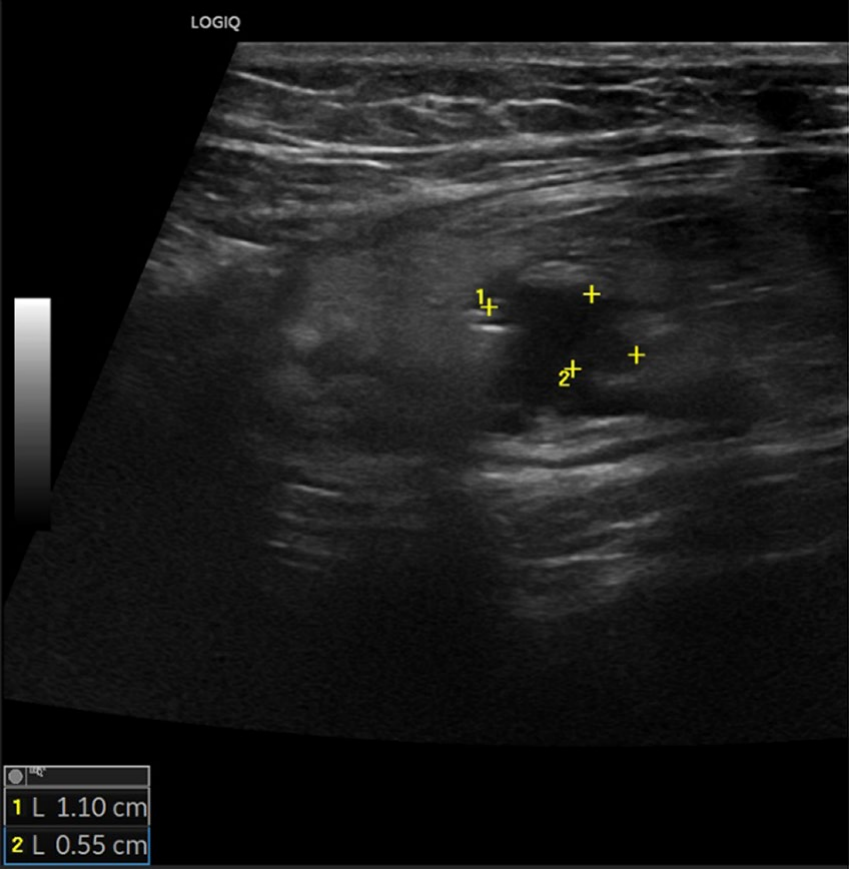
At the level of the descending colon and sigmoid colon, the wall thickness is at the upper limits (3–4 mm), with preserved wall stratification. A micro-perforated diverticulum is observed, accompanied by a small 11 mm collection and a hyperechoic appearance of the adjacent adipose tissue

**Fig. 7:**
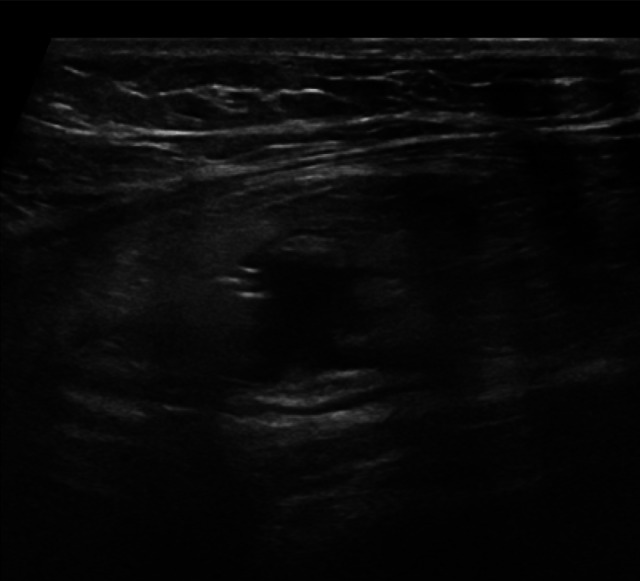
At the level of the descending colon and sigmoid colon, the wall thickness is at the upper limits (3–4 mm), with preserved wall stratification. A micro-perforated diverticulum is observed, accompanied by a small 11 mm collection and a hyperechoic appearance of the adjacent adipose tissue

In cases of acute diverticulitis, the exam can start at the point of maximum tenderness, using the graded compression technique. Alternatively, a standardized approach can be used, which involves locating the sigmoid colon in a transverse section, where it appears ventral to the left iliac artery, and then following it from its distal portion to the proximal portion up to the descending colon [16]. In the context of sigmoid colon diverticulosis, the bowel wall is characterized by a slight thickening of the muscularis propria (the outer hypoechoic layer) [16]. Typically, in this setting, diverticula appear on ultrasound as outpouchings of the wall containing hyperechoic material, represented by gaseous interfaces, feces, or fecaliths, and are characterized by acoustic shadowing [29] (Fig. 3, 4 and 5). In cases of diverticulitis, the following ultrasound signs are frequently observed: segmental thickening of the colonic wall (> 5 mm), evidence of the inflamed diverticulum in the thickened wall area, and changes in the pericolic tissue, which appears non-compressible and hyperechoic [16, 30]. The inflamed diverticulum may appear hypoechoic (37% of cases), hyperechoic (4%), hyperechoic with a surrounding hypoechoic rim (41%), or hyperechoic with acoustic shadowing (18%) [31]. Around the diverticulum, hyperechoic, non-compressible tissue is observed, representing the inflamed mesentery and omentum [30] (Figs. 6 and 7). In complicated cases, typical ultrasound findings include, in milder cases, pericolic air bubbles or a small pericolic fluid collection. In more severe cases, a peri-diverticular abscess smaller or larger than 4 cm, gas more than 5 cm away from an inflamed diverticulum, or, in cases of persistent colon perforation, diffuse fluid collection with distant free air can be visualized [32]. On ultrasound, contained perforations, fistulas, and abscesses are characterized by air bubbles in the mesentery or within a hypoechoic fluid collection. Specifically, fistulas can involve an adjacent intestinal loop, the bladder, or the uterus, and appear on ultrasound as hypoechoic bands with or without central gas bubbles [16]. The presence of free air in the peritoneum or air bubbles in the retroperitoneal space indicates a free or retroperitoneal perforation [16].

## Systematic review of recent evidence

A recent systematic review and meta-analysis (12 studies; *n* = 2,056) found that ultrasound (US) has high overall diagnostic accuracy for acute diverticulitis when benchmarked against CT (Table 2): sensitivity 92.5% (95% CI 86.9–95.8), specificity 87.7% (75.7–94.2), LR + 8.28 (4.74–14.45), LR − 0.08 (0.05–0.15) [33]. Subgroup analyses showed higher sensitivity with (POCUS)—94.1% (91.4–95.9) and specificity 89.8% (77.6–95.7)—compared with radiology-performed US — sensitivity 83.2% (68.3–91.9), specificity 88.7% (76.1–95.1) [33]. For complicated diverticulitis, pooled performance demonstrated lower sensitivity (58.3% [46.1–69.8]) but very high specificity (98.2% [96.4–99.2]), with LR + 31.86 (15.61–65.06) and LR − ~ 0.42 (0.32–0.56) [33]. Complementary primary studies refine these findings. In a multicenter Asian cohort of uncomplicated disease (*n* = 326), POCUS achieved overall accuracy 92% (95% CI 89.1–95.0), but performance varied by location, being lower in cecal diverticulitis (accuracy 84.3%, 77.8–90.8); higher BMI independently reduced accuracy in cecal cases (OR 0.79, 0.64–0.97) [34]. A prospective Italian study (*n* = 55) reported POCUS sensitivity 98% and specificity 88% versus contrast-enhanced CT (CEACT), with Hinchey staging concordance 93% (H1–H3) [35]. In a multicenter ED study integrating clinical assessment with POCUS (*n* = 393), accuracy for diverticulitis was high (sensitivity 92.7%, specificity 90.9%), time-to-diagnosis fell markedly (≈97 vs 330 min), and a POCUS-guided triage strategy would have sent ~ 49% for CT while achieving 94% sensitivity for detecting complicated disease via CT selection; however, POCUS alone had low sensitivity (50%) for complicated diverticulitis [36]. Therefore, POCUS can rapidly and accurately confirm suspected diverticulitis and reduce time to disposition. For complicated diverticulitis, US remains highly specific but insufficiently sensitive; thus, CT is recommended when complications are suspected or when POCUS is equivocal, with awareness of anatomic (cecal) and patient (BMI) factors that may degrade performance.

**Table 2:** Summary of POCUS diagnostic accuracy studies for acute diverticulitis

Study	Year	Design/setting	Population	Condition	N (studies/patients)	Sensitivity (95% CI)	Specificity (95% CI)	LR + (95% CI)	LR − (95% CI)	Accuracy (95% CI)	Notes
Shokoohi et al. [33]	2025	Systematic review & meta-analysis	Adults with suspected acute diverticulitis	Overall diverticulitis	Subgroup across studies	94.1% (91.4–95.9)	89.8% (77.6–95.7)	–	–	–	Higher sensitivity at bedside vs. radiology
Huang et al. [34]	2023	Multicenter, 10-year observational	Adults; Asian cohort; suspected uncomplicated diverticulitis	Uncomplicated diverticulitis (by location)	1/326	–	–	–	–	92% (89.1–95.0); Cecum: 84.3% (77.8–90.8)	9/10 false positives were appendicitis; BMI reduced accuracy in cecal cases (OR 0.79, 0.64–0.97)
Zago et al. [35]	2021	Prospective; surgeons performing POCUS; CE-CT reference	Adults; Italian ED; suspected diverticulitis	Overall diverticulitis; Hinchey staging	1/55	98%	88%	–	–	96%	Hinchey staging concordance 93% (H1–H3) vs CE-CT
Nazerian et al. [36]	2021	Prospective multicenter; clinical + POCUS vs standard care	Adults; ED; suspected diverticulitis	Overall diverticulitis; complicated diverticulitis triage	1/393	92.7% (overall); 50% (complicated, POCUS alone)	90.9% (overall)	–	–	–	Time-to-diagnosis shorter (≈97 vs 330 min, *P* < 0.001); CT triage sensitivity 94% for complicated cases

*ED* Emergency Department, *POCUS* Point-of-Care Ultrasound, *CT* Computed Tomography, *CE*-*CT* Contrast-Enhanced CT, *CI* Confidence Interval, *LR* + Positive Likelihood Ratio, *LR* − Negative Likelihood Ratio, *OR* Odds Ratio, *BMI* Body Mass Index.

## Inflammatory bowel disease

**Crohn’s Disease**

**Fig. 8:**
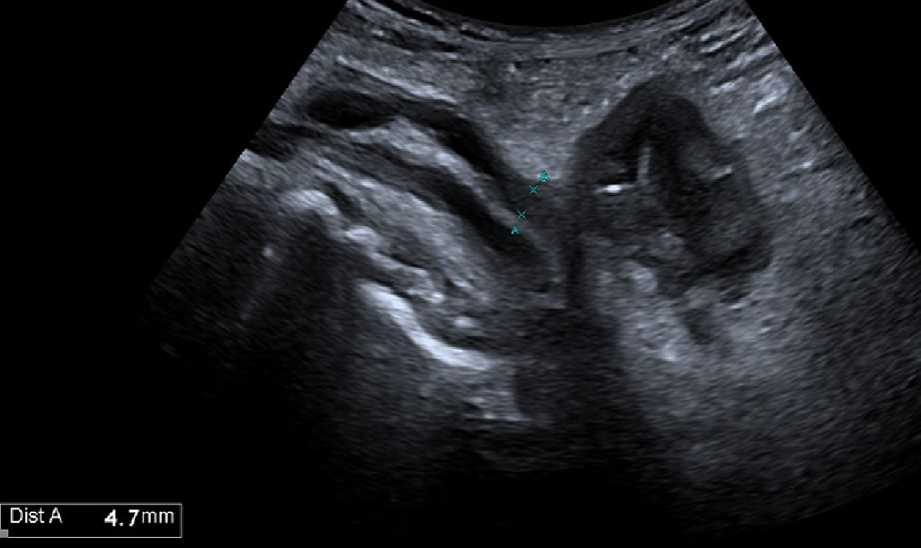
In the right iliac fossa, the final loop of the ileum shows, over a segment of 7 cm, thickened walls measuring 5 mm, with preserved stratification and the presence of vascular signals in the walls as detected by color Doppler imaging (Limberg 3). Another segment, located more proximally in the ileum and observed in the hypogastric region, measures approximately 5 cm, with walls thickened up to 10 mm, loss of stratification, and the presence of vascular signals in the walls on color Doppler imaging (Limberg 4). These segments show a narrowed lumen and dilation of the upstream bowel loop (55 mm diameter)

**Fig. 9:**
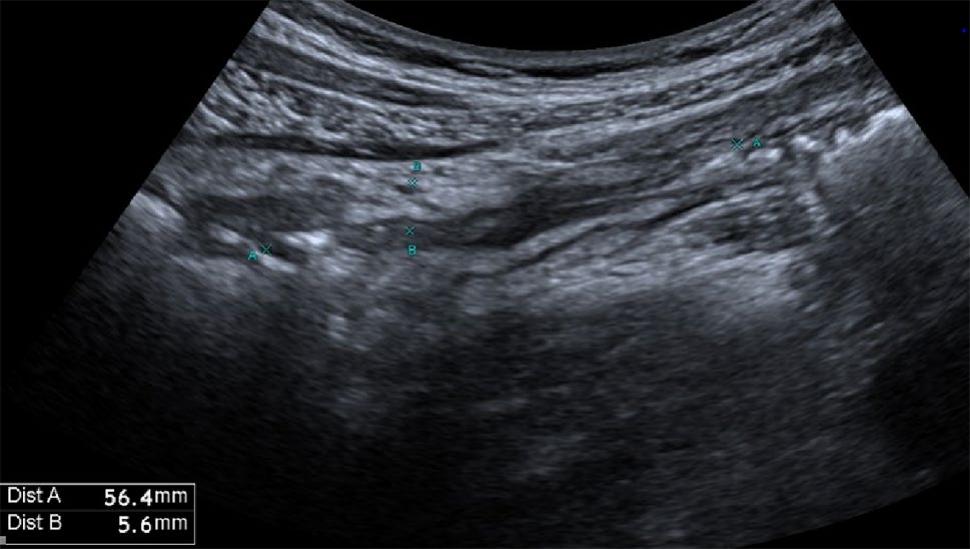
In the right iliac fossa, the final loop of the ileum shows, over a segment of 7 cm, thickened walls measuring 5 mm, with preserved stratification and the presence of vascular signals in the walls as detected by color Doppler imaging (Limberg 3). Another segment, located more proximally in the ileum and observed in the hypogastric region, measures approximately 5 cm, with walls thickened up to 10 mm, loss of stratification, and the presence of vascular signals in the walls on color Doppler imaging (Limberg 4). These segments show a narrowed lumen and dilation of the upstream bowel loop (55 mm diameter)

**Fig. 10:**
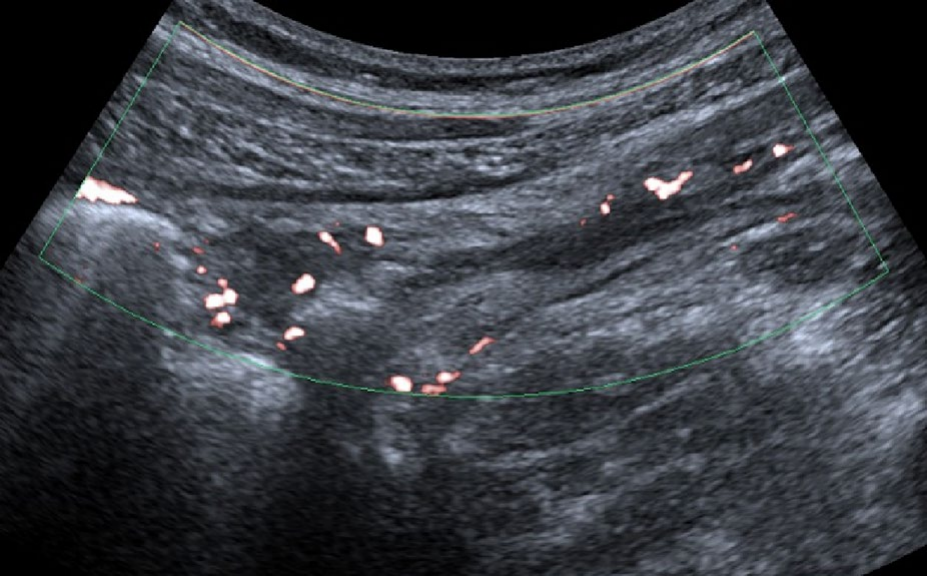
In the right iliac fossa, the final loop of the ileum shows, over a segment of 7 cm, thickened walls measuring 5 mm, with preserved stratification and the presence of vascular signals in the walls as detected by color Doppler imaging (Limberg 3). Another segment, located more proximally in the ileum and observed in the hypogastric region, measures approximately 5 cm, with walls thickened up to 10 mm, loss of stratification, and the presence of vascular signals in the walls on color Doppler imaging (Limberg 4). These segments show a narrowed lumen and dilation of the upstream bowel loop (55 mm diameter)

**Fig. 11:**
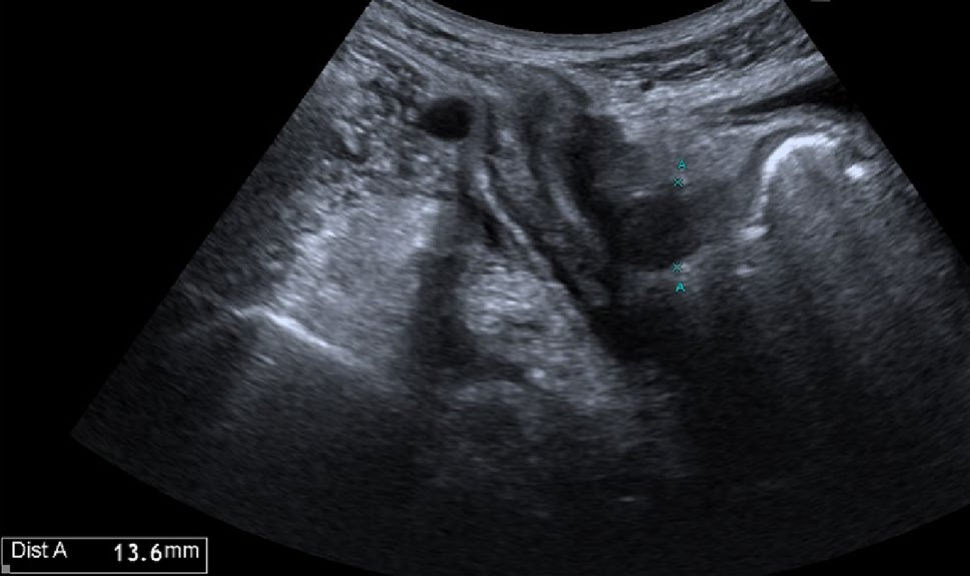
In the right iliac fossa, the final loop of the ileum shows, over a segment of 7 cm, thickened walls measuring 5 mm, with preserved stratification and the presence of vascular signals in the walls as detected by color Doppler imaging (Limberg 3). Another segment, located more proximally in the ileum and observed in the hypogastric region, measures approximately 5 cm, with walls thickened up to 10 mm, loss of stratification, and the presence of vascular signals in the walls on color Doppler imaging (Limberg 4). These segments show a narrowed lumen and dilation of the upstream bowel loop (55 mm diameter)

**Fig. 12:**
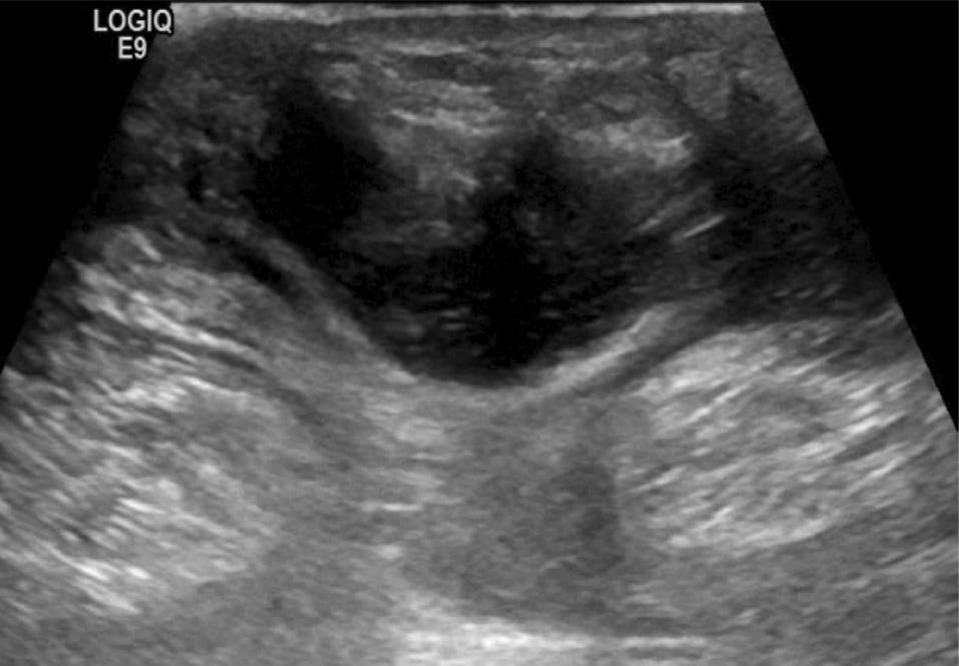
At the site of the cutaneous fistulous opening, an elongated entero-mesenteric collection with a fistulous tract is visualized. This collection extends anteriorly, just beneath the adipose tissue, and becomes superficial, reaching as close as 7 mm from the skin surface

**Fig. 13:**
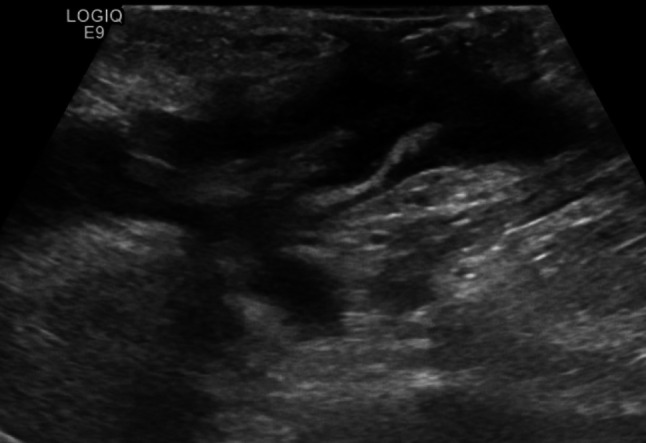
At the site of the cutaneous fistulous opening, an elongated entero-mesenteric collection with a fistulous tract is visualized. This collection extends anteriorly, just beneath the adipose tissue, and becomes superficial, reaching as close as 7 mm from the skin surface

Bowel wall thickening is one of the main ultrasound findings in Crohn’s disease (CD). A threshold value of > 3 mm is considered pathological, with a sensitivity of 89% and a specificity of 96%. For values exceeding 4 mm, sensitivity and specificity are 87% and 98%, respectively [37] (Figs. 8, 9, 10 and 11). Strictures, abscesses, phlegmons, and fistulas are common complications. Fistulas are connections between the lumens of adjacent organs (entero-enteric, entero-vesical, entero-mesenteric, entero-vaginal) or between the lumen of a bowel loop and the skin (entero-cutaneous). On ultrasound, they appear as hypoechoic areas connecting two adjacent bowel loops, with or without internal gas artifacts [38]. Fistulas can lead to abscesses, which are purulent collections that, in B-mode imaging, appear as hypoechoic to anechoic areas with irregular margins, posterior wall enhancement, and fluid content with possible gaseous components [39, 40] (Figs. 12 and 13). However, magnetic resonance imaging (MRI) is the method of choice for detecting a fistula. An ultrasound finding of a hypoechoic lesion with poorly defined, blurred margins and no evidence of liquefaction is suggestive of a phlegmon. Strictures are the primary cause of intestinal obstruction, which on ultrasound appears as dilation (> 25–30 mm) and hyperperistalsis of the upstream segment proximal to the stenotic bowel, which shows thickened walls and a narrowed lumen (diameter less than 1 cm). This is often accompanied by the accumulation of fluid and/or gas [39, 40]. Although multiple studies reported high sensitivity and specificity of bowel ultrasound for detecting inflammatory activity and complications, in routine care its most robust role is in follow-up and treatment monitoring, rather than comprehensive primary mapping—particularly in Crohn’s disease with skip lesions, where endoscopy and/or cross-sectional imaging are often required to assess extent and transmural involvement [41, 42]. Reported accuracies are also strongly influenced by operator experience and standardized acquisition/measurement protocols [43, 44].

**Ulcerative colitis (UC)**

UC is an inflammatory disease that affects only the mucosa of the colon, which can involve the rectum and extend to the entire colon [39]. During a flare-up of UC, ultrasound frequently reveals continuous thickening of the mucosa and submucosa, ranging between 4 and 9 mm [45]. The submucosa often shows increased echogenicity, while the mucosa generally appears irregular due to gas bubbles trapped between pseudo-polyps. Additionally, it may be interrupted by deep ulcerations, and there is a characteristic loss of haustration [46]. A potentially life-threatening complication of this disease that requires prompt treatment is a toxic megacolon, characterized by an abnormal dilation of the colon lumen exceeding 6 cm, accompanied by reduced wall thickness and liquid distension of the intestinal loops [40].

## Systematic review of recent evidence

A single-center feasibility study evaluated POCUS performed by a gastroenterologist to assess IBD activity and complications, using cross-sectional imaging (CT enterography or MR enterography) as the comparator for Crohn’s disease and colonoscopy for ulcerative colitis [47]. Over May 2015–March 2016, 178 BUS exams were performed in patients with suspected or established IBD; 79 had a reference test within 3 months. POCUS demonstrated high sensitivity for key targets: bowel wall thickening (inflammation) 90%, Crohn’s stenosis 94%, and inflammatory mass 75% [47].

## Inguinal and femoral hernias

**Fig. 14:**
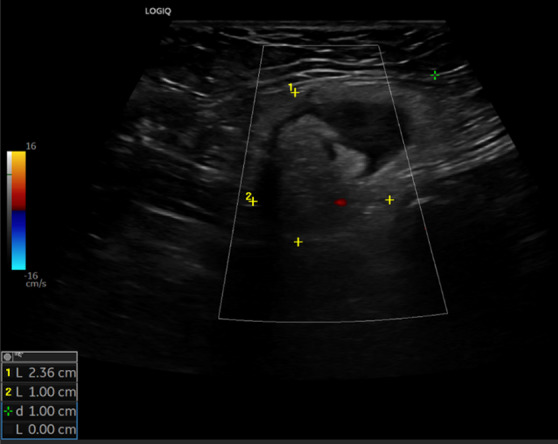
In the left inguinal region, a hernia orifice measuring 10 mm is visible, through which omental fat is protruding, showing weak arterial signals on Doppler imaging. Additionally, a small amount of peri-omental fluid is present

**Fig. 15:**
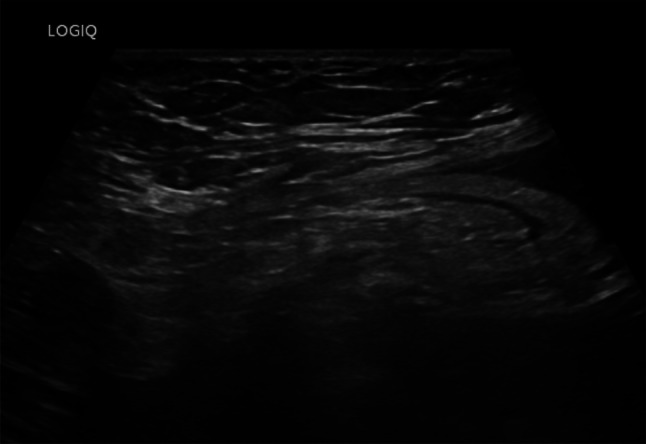
In the left inguinal region, a hernia orifice measuring 10 mm is visible, through which omental fat is protruding, showing weak arterial signals on Doppler imaging. Additionally, a small amount of peri-omental fluid is present

The standardized method for identifying an inguinal hernia involves scanning the ilioinguinal crease and adjacent areas just above and below the inguinal line. Alternatively, the point of maximum tenderness can be used to locate the hernia. To distinguish between direct and indirect inguinal hernias, the inguinal canal is scanned near the inferior epigastric vessels [48]. If it is difficult to visualize the hernia, the patient can be asked to perform the Valsalva maneuver or the examination can be performed with the patient standing [49]. Inguinal and femoral hernias are diagnosed by visualizing on US omental fat or intestinal contents through a defect in the abdominal wall. Fat appears hyperechoic, while intestinal contents typically exhibit mixed echogenicity due to fecal material, fluid, or gas within the lumen [48]. Inguinal hernias are often occupied by omental fat and/or intestinal structures, the latter distinguishable by the presence of peristalsis, circular folds in the mucosal layer of the small intestine, and haustral folds in the colon [50] (Figs. 14 and 15). The most common complications of inguinal hernias are incarceration and strangulation. Incarcerated hernias are characterized by the absence of peristalsis, the presence of surrounding free fluid, preserved blood flow on color Doppler, or contrast-enhanced ultrasound (CEUS) [51]. and the inability to reduce the incarcerated loop with constant probe pressure [52]. In such cases, small bowel obstruction (SBO) is suspected when the following ultrasound findings are observed: dilated fluid-filled intestinal loops (diameter > 2.5 cm) throughout the abdomen, altered peristalsis (“to-and-fro” movement), free fluid between intestinal loops, and persistent blood flow on color or power Doppler applied to the bowel wall [53, 54]. Finally, strangulation is a medical emergency characterized in advanced stages by the absence of flow on color and power Doppler within the mucosal wall, indicative of ischemia. In the early stages, findings include bowel edema, free abdominal fluid, bowel wall thickness greater than 3 mm, and loss of peristalsis [55].

To our knowledge, no original studies have evaluated the use of POCUS for inguinal or femoral hernias.

## Bowel obstruction

**Fig. 16:**
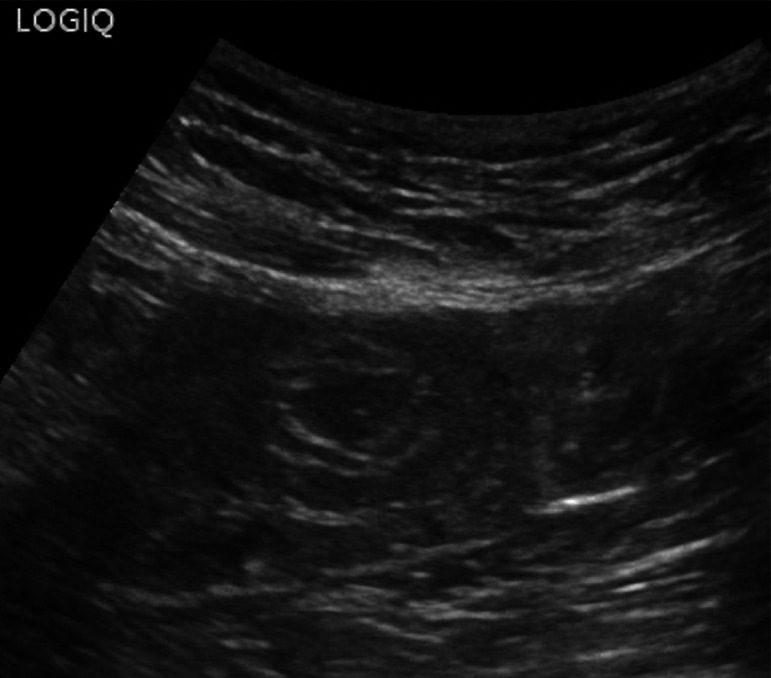
In the left iliac fossa, in transverse scans, a “target” image is observed, with a typical “loop within loop” configuration visible in longitudinal scans, consistent with intestinal intussusception involving the small intestine

**Fig. 17:**
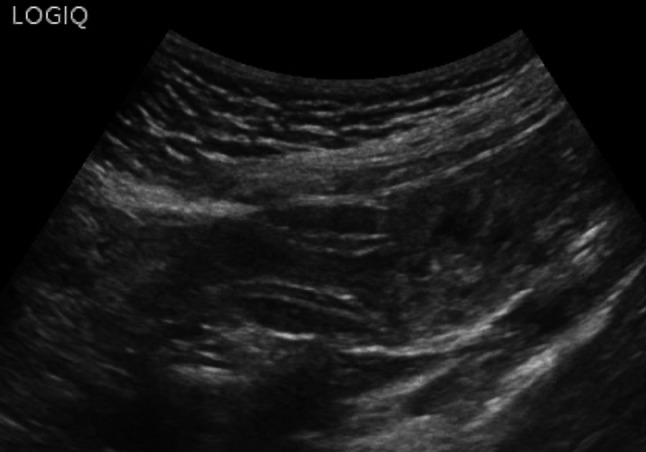
In the left iliac fossa, in transverse scans, a “target” image is observed, with a typical “loop within loop” configuration visible in longitudinal scans, consistent with intestinal intussusception involving the small intestine

**Fig. 18:**
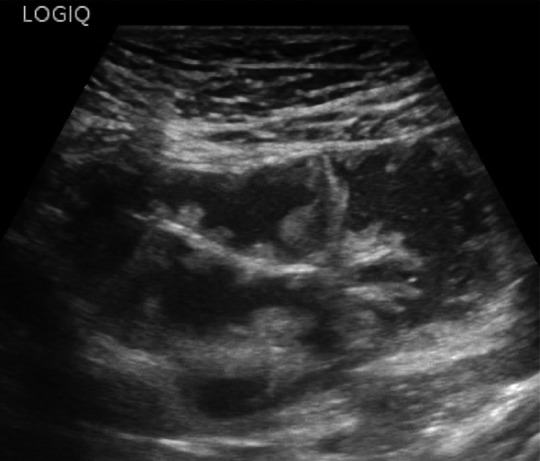
Proximal to the aforementioned intussuscepted intestinal segment, several distended jejunal loops filled predominantly with fluid material are observed

**Fig. 19:**
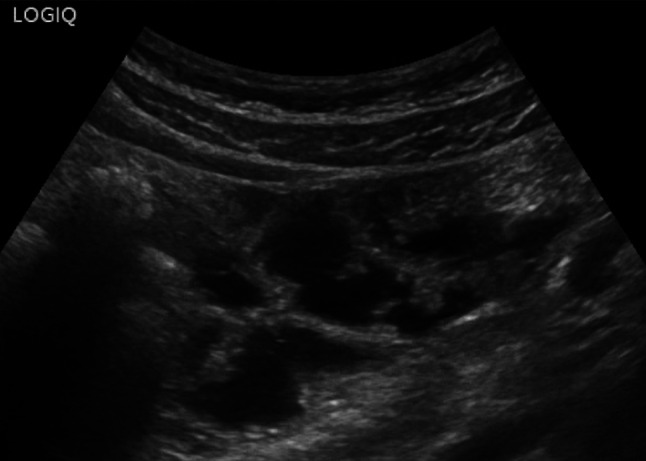
Proximal to the aforementioned intussuscepted intestinal segment, several distended jejunal loops filled predominantly with fluid material are observed

**Fig. 20:**
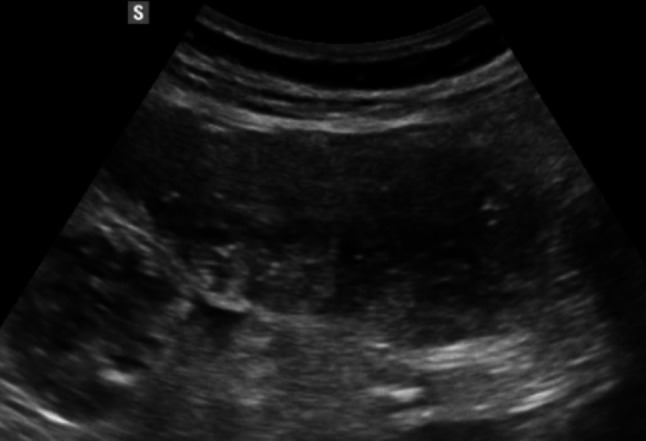
Jejunal loops appear markedly dilated, with a diameter exceeding 2.5 cm and walls of regular thickness, with the lumen filled predominantly with fluid exhibiting the typical “to-and-fro” movement. Additionally, a slight perivisceral fluid layer is observed

**Fig. 21:**
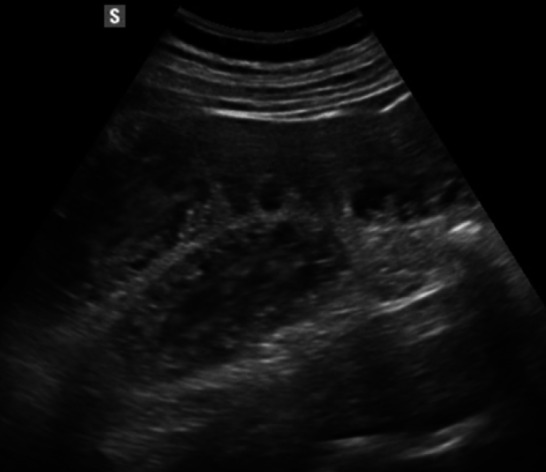
Jejunal loops appear markedly dilated, with a diameter exceeding 2.5 cm and walls of regular thickness, with the lumen filled predominantly with fluid exhibiting the typical “to-and-fro” movement. Additionally, a slight perivisceral fluid layer is observed

**Fig. 22:**
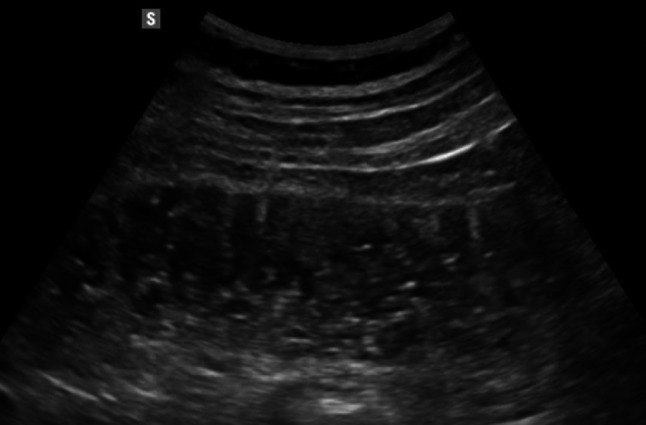
Jejunal loops appear markedly dilated, with a diameter exceeding 2.5 cm and walls of regular thickness, with the lumen filled predominantly with fluid exhibiting the typical “to-and-fro” movement. Additionally, a slight perivisceral fluid layer is observed

Small bowel obstruction can be functional (ileus) or mechanical. A 3-step examination technique was suggested to have an overview of the presence of intestinal obstruction and the segments involved. This consists primarily of scanning the epigastrium to visualize the stomach. In patients with high obstruction it is possible to see the stomach outstretched even via a sub-xiphoidal approach or a trans-lienal view on the gastric fundus [56]. Then the left mid-abdomen is assessed to show the jejunum and descending colon. Finally, a right lower abdomen view enables the evaluation of the ileocecal junction [57]. Paralytic ileus is defined as a distension of the small bowel loops, which appear filled with gas and fluid in the absence of peristalsis, associated with feces, gas, and fluid in the colon [58]. In cases of mechanical bowel obstruction, ultrasound could identify the site of obstruction by following the course of the dilated loops up to the transition point between the proximal dilated loop and the distal collapsed loop, even CT is the gold standard method [59]. On ultrasound, the obstruction appears as dilated bowel loops measuring up to 3 cm in diameter and at least 10 cm in length [56], increased bowel wall thickness exceeding 3 mm [58], increased intestinal contents [56], and increased peristaltic activity (“to-and-fro” movement), which may decrease as the duration of the obstruction increases [59] (Figs. 16, 17, 18, 19, 20, 21 and 22). Enlarged and visible valvulae conniventes (over 2 mm) and a collapsed colonic lumen are also observed [58]. The appearance of the intestinal contents can help differentiate recent or subocclusive forms, where it appears corpusculated, from prolonged forms, where it is anechoic [56]. To identify the site of obstruction, it is possible to evaluate the presence of valvulae conniventes (Kerckring valves) in the dilated segment; these are generally more numerous in the jejunum compared to the ileum [59]. These structures become prominent in jejunal obstructions and absent or rare in ileal obstructions, “Keyboard sign” [58]. Another useful ultrasound finding for detecting the site of obstruction, known as the “feces sign,” is characterized, above the stricture, by the presence of hyperechoic content due to ingested particles mixed with gas [60]. The loss of normal visceral sliding and longitudinal movement of the intra-abdominal organs caused by the respiratory excursions of the diaphragm is suggestive of adhesions [61]. In cases of intestinal intussusception, the invaginated loop within the invaginating loop can be identified in longitudinal scans (Fig. 17), known as the “sandwich sign” or “fork sign” [59, 62]. In transverse scans (Fig. 16), the “doughnut sign,” characterized by a thickened hypoechoic outer layer with a central hyperechoic core, and the “multiple concentric rings sign,” appearing as a mass with alternating hypoechoic and hyperechoic concentric rings, can be observed [59, 62]. The presence of intraperitoneal free fluid, bowel wall thickening > 4 mm, reduced or absent peristalsis, and reduced or absent bowel wall perfusion on Doppler ultrasound are suggestive of complications. In advanced stages, the presence of free intraperitoneal gas indicates intestinal perforation [59].

**Fig. 23:**
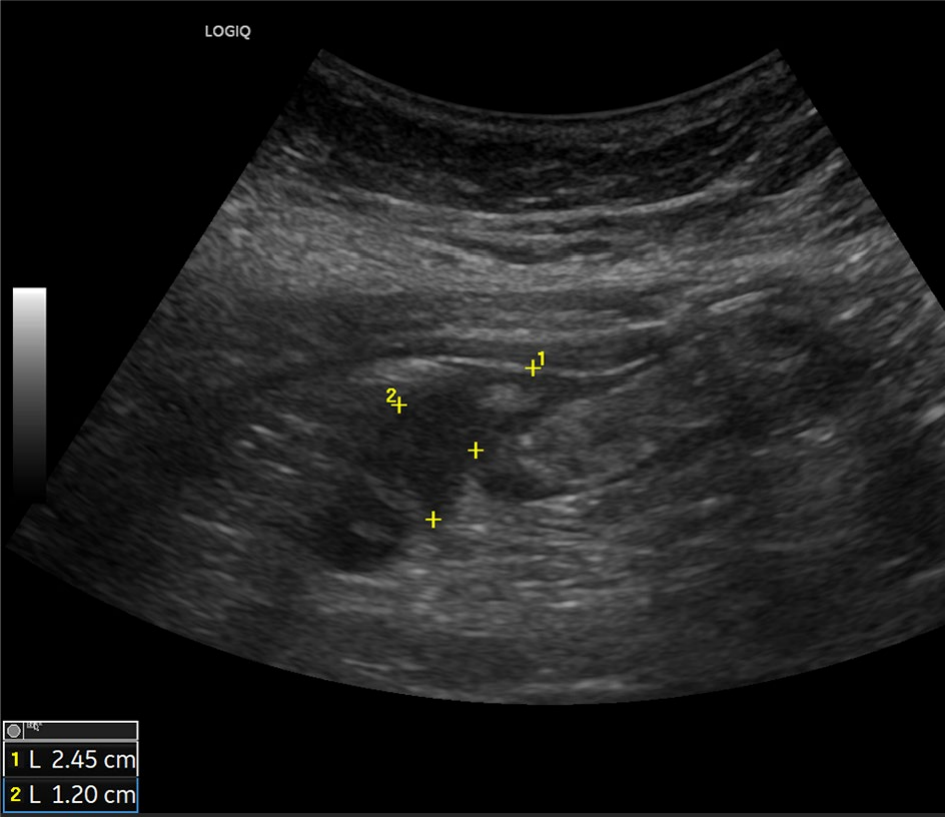
Posterolateral to the ascending colon, a perivisceral fluid collection is observed with transverse dimensions of 25 × 12 mm and a maximum cranio-caudal extension of 5 cm. Within this collection, an iso-hypoechoic outpouching without thickened walls is evident, a finding consistent with epiploic appendagitis

**Large bowel obstruction** (LBO) is characterized on ultrasound by a dilated segment of the colon measuring more than 4.5 cm upstream of the obstruction and a nondilated segment downstream [56]. Liquid content is often observed in the lumen of the right colon, while solid stools are typically present in the left colon [59]. In adults, colorectal cancer is the first cause of LBO, which accounts for 60% of cases, followed by volvulus and diverticulitis, which, when complicated by inflammatory bowel adhesions, pericolic fibrosis and large abscesses, represent approximately 10–15% and 10% of cases, respectively [63]. Extracolonic diseases, through extrinsic compression of the bowel due to peritoneal malignancy, direct invasion, or lymphatic or hematogenous metastasis, account for 10% of LBO cases [63]. An increase in the diameter of the large intestine > 6 cm, the presence of abdominal A-lines, abnormal bowel peristalsis, and thickening of the plicae circulares > 2 mm are the main ultrasound criteria for the diagnosis of LBO [56].

## Systematic review of recent evidence

Evidence supports POCUS as a useful front-end test and operational accelerator in the ED (Table 3). A single-center retrospective study (n = 106) found that adding POCUS was associated with markedly shorter time to diagnosis (median 121 vs 217 min, *P* < 0.001), reduced ED processing time (276 vs 376 min, *p* = 0.006) and ED length of stay (333 vs 436 min, *P* = 0.010), and lower abdominal radiography rates (49% vs 78%, *P* = 0.004); hospital length of stay (LOS) was similar and an observed mortality difference (0 vs 5 deaths) was not statistically significant (*P* = 0.063) [64]. A previous prospective observational study evaluated ED patients with suspected SBO, with a blinded physician-sonographers performing POCUS using a prespecified case definition—bowel-loop diameter ≥ 25 mm with abnormal peristalsis—and assessed ancillary signs (maximum dilatation, visible peristalsis, intraluminal free fluid, bowel wall thickness) [53]. Among 125 patients (median age 54 y; 46% male), 32 (25.6%) had SBO and 9 (7.2%) underwent surgery. POCUS demonstrated sensitivity 87.5% (95% CI 71.0–96.5), specificity 75.3% (65.2–83.6), and AUC 0.74 (0.66–0.82), with similar performance across physician training levels [53]. Operationally, POCUS markedly shortened time to imaging result (~ 11 min) versus abdominal radiography (~ 1 h 38 min) and CT (~ 3 h 42 min) [53].

**Table 3:** Studies evaluating point‑of‑care ultrasound for bowel obstruction

Study	Year	Design/setting	*N*	Sensitivity (95% CI)	Specificity (95% CI)	LR + (95% CI)	LR − (95% CI)	Accuracy (95% CI)	Comparator	Key outcomes/notes
Di Gioia et al. [64]	2024	Retrospective, single-center ED	106	–	–	–	–	–	CT confirmation in all; operational outcomes	Time to Dx ↓ (121 vs 217 min, p < 0.001); ED processing ↓ (276 vs 376 min, p = 0.006); ED LOS ↓ (333 vs 436 min, p = 0.010); fewer X-rays (49% vs 78%, p = 0.004); hosp LOS similar; mortality 0 vs 5 (p = 0.063)
Shokoohi et al. [33]	2023	IPD meta-analysis of 5 prospective studies	433	83.0% (71.7–90.4)	93.0% (55.3–99.3)	11.9 (1.2–114.9)	0.2 (0.1–0.3)	0.88 (95% CI 0.85–0.91)	Final Dx during hospitalization	Attendings: sens 87.7%, spec 91.4; Residents: sens 73.0%, spec 88.2; BMI ≥ 30: sens 72.0%, spec 89.5 (vs BMI < 30: sens 88.6%, spec 84.0)
Boniface et al. [53]	2020	Prospective observational ED study (physician sonographers blinded)	125	87.5% (71.0–96.5)	75.3% (65.2–83.6)	–	–	AUC 0.74 (0.66–0.82)	CT (reference standard); timing vs CT and abdominal radiography	Time to result: POCUS ~ 11 min vs X-ray ~ 1 h 38 min vs CT ~ 3 h 42 min. Performance similar across training levels
Becker et al. [66]	2019	Prospective, multicenter ED	217	88% (80–94)	54% (45–63)	1.92 (1.56–2.35)	0.22 (0.12–0.39)	–	Expert blinded review of same clips: sens 89%, spec 82%	Most sensitive signs: SB dilation ≥ 25 mm, abnormal peristalsis; Most specific: transition point, free fluid, wall edema

*POCUS* Point‑of‑Care Ultrasound, *ED* Emergency Department, *LOS* Length of Stay, *CT* Computed Tomography, *CI* Confidence Interval, *LR* + Positive Likelihood Ratio, *LR* − Negative Likelihood Ratio, *BMI* Body Mass Index

An individual patient-level meta-analysis of 5 prospective studies (*n* = 433) reported sensitivity 83.0% (95% CI 71.7–90.4) and specificity 93.0% (55.3–99.3) for POCUS diagnosis of SBO (LR + 11.9 [1.2–114.9]; LR − 0.2 [0.1–0.3]). Diagnostic performance was lower for residents (sens 73.0% [56.6–84.9], spec 88.2% [58.8–97.5]) than attendings (sens 87.7% [71.1–95.4], spec 91.4% [57.4–98.8]), and declined with BMI ≥ 30 kg/m^2^ (sens 72.0% [50.6–87.9], spec 89.5% [75.2–97.1]) versus < 30 (sens 88.6% [79.5–94.7], spec 84.0% [75.3–90.6]) [65]. A multicenter ED study (*n* = 217) showed POCUS sensitivity 0.88 (95% CI 0.80–0.94) but specificity 0.54 (0.45–0.63) by bedside emergency physicians; blinded expert review of the same cine loops improved specificity to 0.82 (0.74–0.88) [66]. Feature-level analysis suggested dilated small bowel ≥ 25 mm and abnormal peristalsis were the most sensitive signs (≈0.82–0.87), while transition point, free fluid, and bowel wall edema were more specific (up to 0.98) [66].

Several works have been published about intussusception, especially in children (Table 4). Diagnostic performance of POCUS is consistently excellent. A 2022 meta-analysis (11 studies; *n* = 2,400) found sensitivity 95.1% (90.3–97.2) and specificity 98.1% (95.8–99.2) (LR + 50 [23–113]; LR − 0.05 [0.03–0.09]) [67]. A multicenter non-inferiority study (*n* = 256; 17 sites) reported POCUS accuracy 97.7% (94.9–99.0) vs RADUS 99.3% (96.8–99.9), difference 1.5 percentage points (95% CI − 0.6 to 3.6); sensitivity 96.6%, specificity 98.0% [68]. Novice PEM physicians also achieved high concordance with radiology US (97%; *κ* = 0.826) with sensitivity 89% and specificity 98% in a single-center cohort (n = 100) [69]. Earlier syntheses echo these results: a 2020 meta-analysis (6 studies; *n* = 1,303) reported sensitivity 94.9%, specificity 99.1% (LR + 105; LR − 0.05) [70], and a broader bivariate meta-analysis (30 studies; *n* = 5249) showed US sensitivity 0.98 and specificity 0.98, with no significant difference between POCUS and radiology-performed US (AUROC 0.95 vs 1.00; *P* = 0.128) [71]. Observational ED data suggest POCUS can also differentiate small bowel–small bowel from ileocolic intussusception, supporting triage by highlighting transient, often self-resolving variants (*κ* = 0.85) [72]. Therefore, when performed by trained clinicians, POCUS is near-definitive for ED diagnosis and triage, with performance approaching radiology US.

**Table 4:** Studies assessing point‑of‑care ultrasound for intussusception

Study	Year	Design/setting	N	Sensitivity (95% CI)	Specificity (95% CI)	LR + (95% CI)	LR − (95% CI)	Accuracy (95% CI)	Comparator	Key outcomes/notes
Lin‑Martore et al. [67]	2022	Systematic review & meta-analysis (11 studies)	2400	95.1% (90.3–97.2)	98.1% (95.8–99.2)	50 (23–113)	0.05 (0.03–0.09)	–	Reference standards per primary studies	High overall accuracy for ED POCUS
Bergmann et al. [68]	2021	Multicenter, noninferiority, paired POCUS vs RADUS	256	96.6% (87.2–99.1)	98.0% (94.7–99.2)	–	–	97.7% (94.9–99.0); RADUS 99.3% (96.8–99.9)	RADUS; noninferiority margin 4 pp	Accuracy difference 1.5 pp (95% CI − 0.6 to 3.6)
Arroyo et al. [69]	2021	Prospective diagnostic concordance (PEM physicians)	100	89% (51–99)	98% (92–100)	40.44 (10.07–162.36)	0.11 (0.02–0.72)	–	RADUS (*κ* = 0.826; 97% agreement)	High concordance with radiology US
Lin‑Martore et al. [70]	2020	Systematic review & meta-analysis (6 studies)	1303	94.9% (89.9–97.5)	99.1% (94.7–99.8)	105 (18–625)	0.05 (0.03–0.10)	–	Reference standards per primary studies	ED physician-performed POCUS
Tsou et al. [71]	2019	Systematic review & bivariate meta-analysis (30 studies)	5249	98% (96–98)	98% (95–99)	43.8 (18.0–106.7)	0.03 (0.02–0.04)	AUROC 0.99 (0.98–1.00)	POCUS vs RADUS (no significant difference)	Meta-regression AUROC POCUS 0.95 vs RADUS 1.00 (*P* = 0.128)
Park et al. [72]	2019	Retrospective cohort (ED POCUS)	37	–	–	–	–	–	RADUS/clinical course	POCUS differentiated SB–SB vs ileocolic; *κ* = 0.85; SB–SB often transient

*POCUS* Point‑of‑Care Ultrasound, *RADUS* Radiology‑performed Ultrasound, *ED* Emergency Department, *CI* Confidence Interval, *LR* + Positive Likelihood Ratio, *LR* − Negative Likelihood Ratio, *AUROC* Area Under the ROC Curve, *κ* Cohen’s kappa, *pp* percentage points

## Acute epiploic appendagitis

**Fig. 24:**
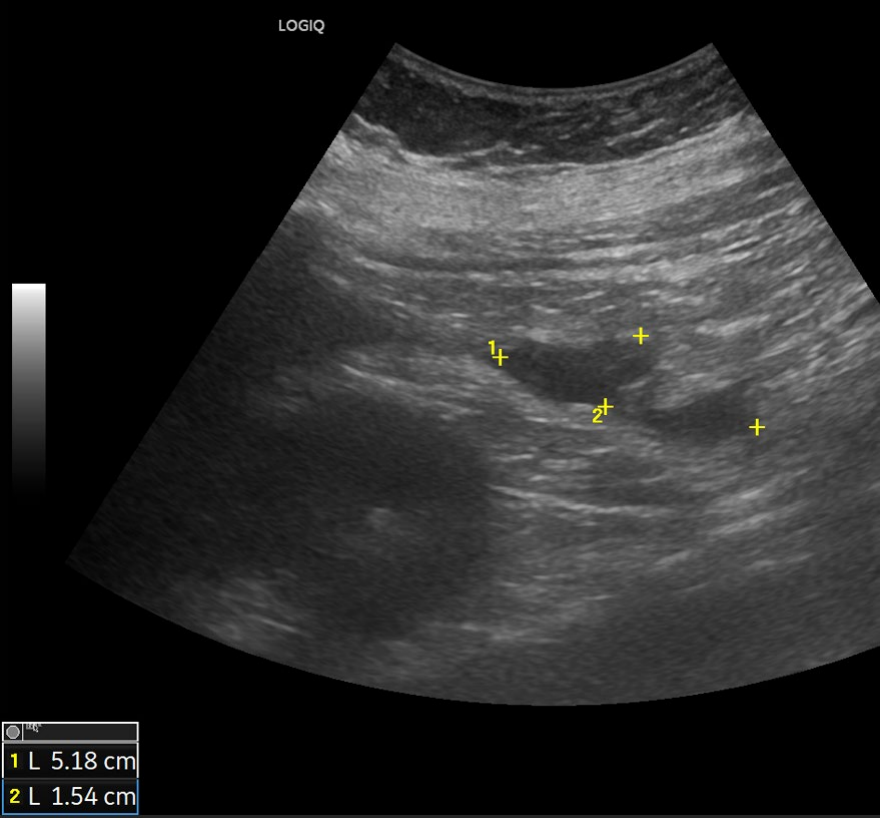
Posterolateral to the ascending colon, a perivisceral fluid collection is observed with transverse dimensions of 25 × 12 mm and a maximum cranio-caudal extension of 5 cm. Within this collection, an iso-hypoechoic outpouching without thickened walls is evident, a finding consistent with epiploic appendagitis

**Fig. 25:**
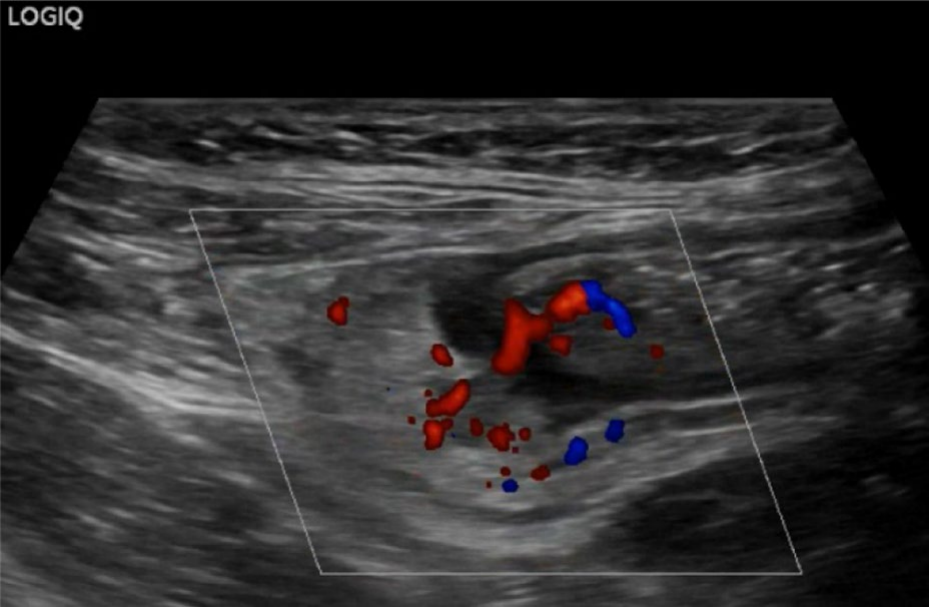
At the level of the right colon, just before the cecum, there is a hypoechoic outpouching measuring 1 cm, with a thickened wall (5 mm) and abundant vascularization on color Doppler imaging

**Fig. 26:**
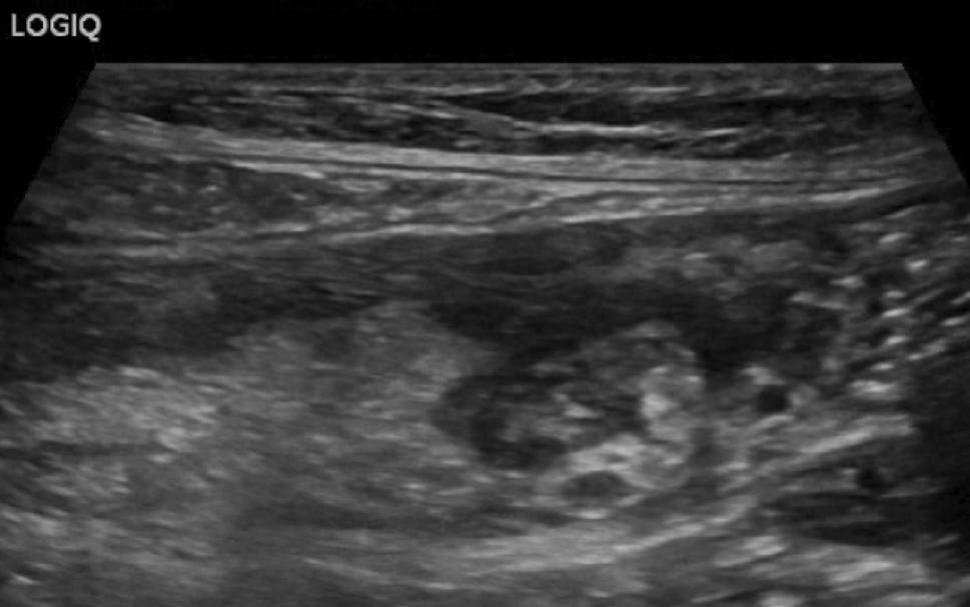
At the level of the right colon, just before the cecum, there is a hypoechoic outpouching measuring 1 cm, with a thickened wall (5 mm) and abundant vascularization on color Doppler imaging

Epiploic appendages are small peritoneal pouches (0.5 ± 5 cm in size) attached to the serous surface of the colon (from the cecum to the rectosigmoid junction), containing fat and small blood vessels (73, 74). Primary acute epiploic appendagitis is a torsion of cecal appendages with consequent edema and potential ischemic necrosis and aseptic inflammation [75]. Epiploic appendagitis is defined as secondary when the inflamed appendage reflects an inflammatory process located in its proximity such as colonic diverticulitis, appendicitis, or cholecystitis [76]. On ultrasound, acute epiploic appendagitis appears as a small, non-compressible hyperechoic ovoid mass adhered to the colonic wall with a lack of central blood flow on Doppler US (Figs. 23, 24, 25 and 26) or CEUS [74, 76]. It is often surrounded by a hypoechoic halo at the point of maximum tenderness [73].

To our knowledge, no original studies have evaluated the use of POCUS for acute epiploic appendagitis.

## ischemic bowel disease

### Acute arterial mesenteric ischemia

Bowel ischemia is classified into acute mesenteric ischemia (small bowel ischemia), venous mesenteric ischemia, non-occlusive mesenteric ischemia, and ischemic colitis [77]. This clinical condition can be caused by arterial embolism or thrombosis, atherosclerosis or venous thrombosis, low cardiac output, permeable vessels, and vasculitis in younger patients [78].

**Fig. 27:**
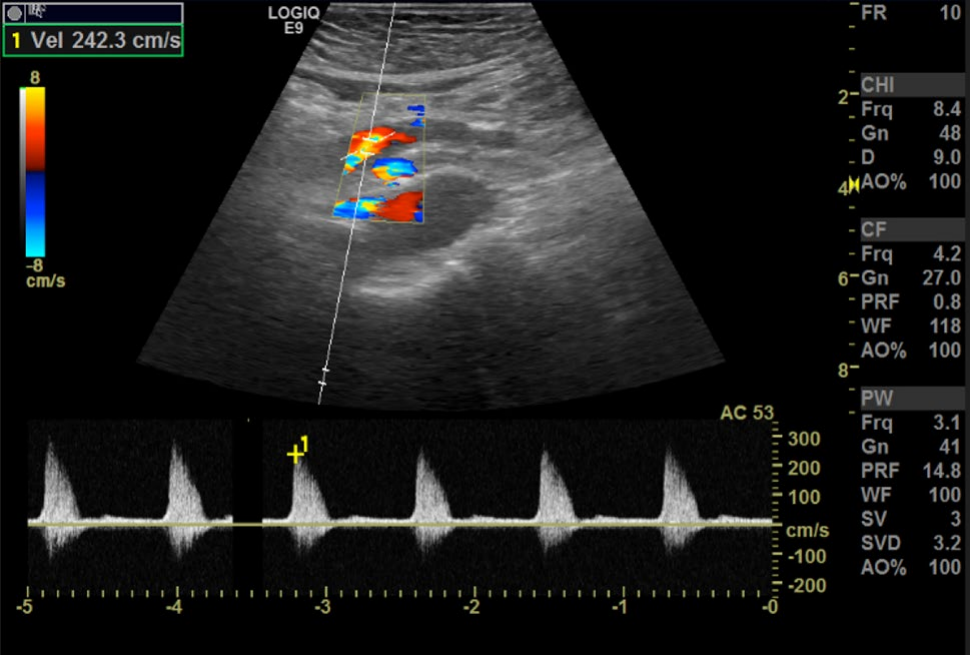
These images show an ectasia of the mesenteric artery approximately 1.5 cm from its origin, with dimensions of 11 × 10 mm (AP x LL). Within the ectatic segment, an eccentric hypoechoic thrombus is visible along the inferior and lateral walls, with a maximum thickness of 6 mm. The thrombus extends along the course of the artery for approximately 2 cm, causing, at its thickest point, a stenosis of about 50–60%

**Fig. 28:**
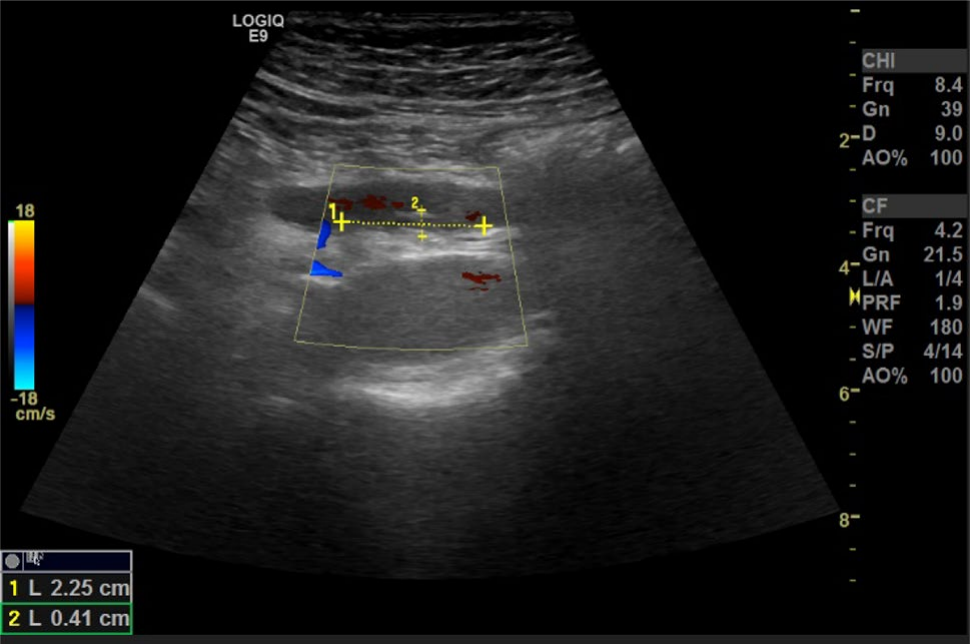
These images show an ectasia of the mesenteric artery approximately 1.5 cm from its origin, with dimensions of 11 × 10 mm (AP x LL). Within the ectatic segment, an eccentric hypoechoic thrombus is visible along the inferior and lateral walls, with a maximum thickness of 6 mm. The thrombus extends along the course of the artery for approximately 2 cm, causing, at its thickest point, a stenosis of about 50–60%

**Fig. 29:**
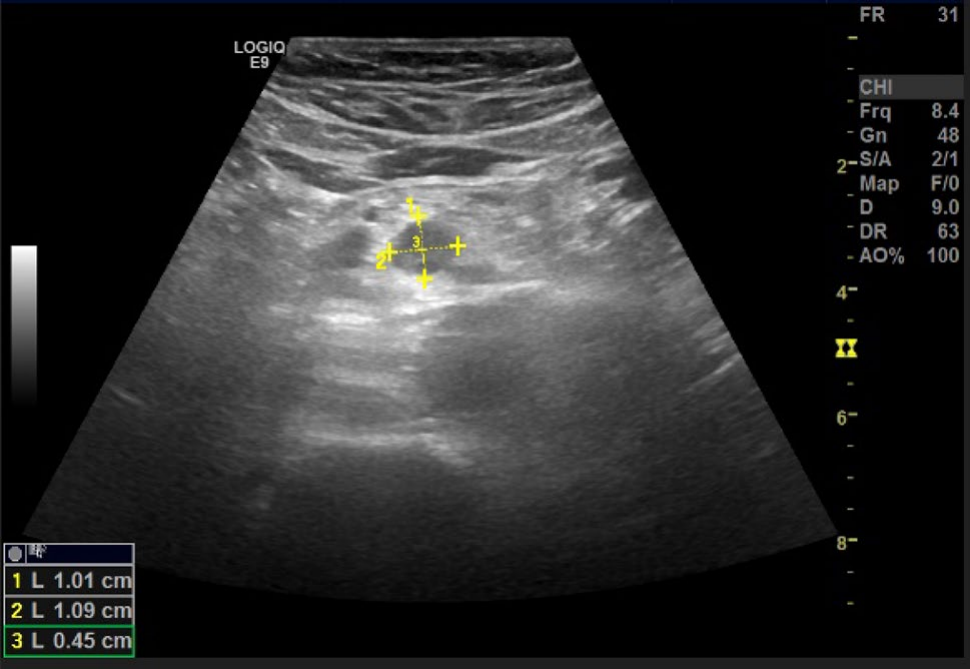
These images show an ectasia of the mesenteric artery approximately 1.5 cm from its origin, with dimensions of 11 × 10 mm (AP x LL). Within the ectatic segment, an eccentric hypoechoic thrombus is visible along the inferior and lateral walls, with a maximum thickness of 6 mm. The thrombus extends along the course of the artery for approximately 2 cm, causing, at its thickest point, a stenosis of about 50–60%

The ultrasound signs suggestive of acute arterial mesenteric ischemia are: thickening of the intestinal wall, decreased peristalsis and increased intraluminal secretions within the involved segments, as well as peritoneal fluid [59, 79, 80]. In cases of obstruction at the origin of the superior mesenteric artery, color Doppler may show the absence of flow in the vessel [13, 81–83]. The presence of color flow in the proximal part of the vessel does not exclude the occlusion of the distal portions of the mesenteric vessels (Figs. 27, 28 and 29) [59]. In acute transmural small bowel infarction of arterio-occlusive origin, the necrotic bowel may typically show a “paper-thin wall” [81]. The final stage of bowel infarction is characterized by late signs: pneumatosis intestinalis and gas in the portal vein [59, 84].

**Non-occlusive mesenteric ischemia** (NOMI) is usually caused by primary mesenteric arterial vasoconstriction [40, 85]. Risk factors for the development of NOMI include coronary artery disease and hypovolemic, septic, and cardiogenic shock [86]. On abdominal ultrasound, the walls of the ischemic colon appear thickened, hypoechoic, and with altered stratification [59]. In the acute phase, there are few color Doppler signals on the wall, while moderate hypervascularization may be present after bowel reperfusion [59].

On ultrasound, **Acute mesenteric venous ischemia is characterized by** a homogeneously hypoechoic thickening of the intestinal wall of the affected segment due to edema and mucosal bleeding, decreased peristalsis, intraluminal secretions, and peri-enteric free fluid can be appreciated [87, 88]. In cases of intestinal ischemia caused by occlusion of the mesenteric veins, the thickening of the intestinal wall is more pronounced compared to cases caused exclusively by occlusion of the mesenteric arteries [87, 89].

**Ischemic colitis** (IC) is a pathological condition caused by an acute or chronic reduction in blood perfusion to the colon, either of occlusive or non-occlusive origin, leading to various degrees of ischemia and potentially causing significant morbidity and mortality [59]. This disorder is classified into two types: gangrenous with transmural necrosis, the most severe given higher associated morbidity and mortality (10–20% of the cases) and non-gangrenous, which is characterized by reversible segmental involvement of the mucosa or submucosa, which benefits from conservative treatment [90]. It typically shows circumferential hypoechoic thickening of the bowel wall greater than 3 mm, segmental (greater than 10 cm) colonic involvement, variable loss of mural stratification, hyperechoic pericolonic fat enhancement, pneumatosis and/or the presence of free fluid, and abrupt transition from the ischemic to the normal bowel segment [91]. Color Doppler flow may be absent or diminished in the bowel wall, especially in the initial phase [59]. In reversible cases, blood flow can be detected on Doppler US and it represents reperfusion of the gut wall, as a result of the resolution of ischemia [91]. The finding of changes in the peri-enteric fat has been related to transmural necrosis [91].

To our knowledge, no original studies have evaluated the use of POCUS for ischemic bowel diseases.

## Bowel perforation

**Fig. 30:**
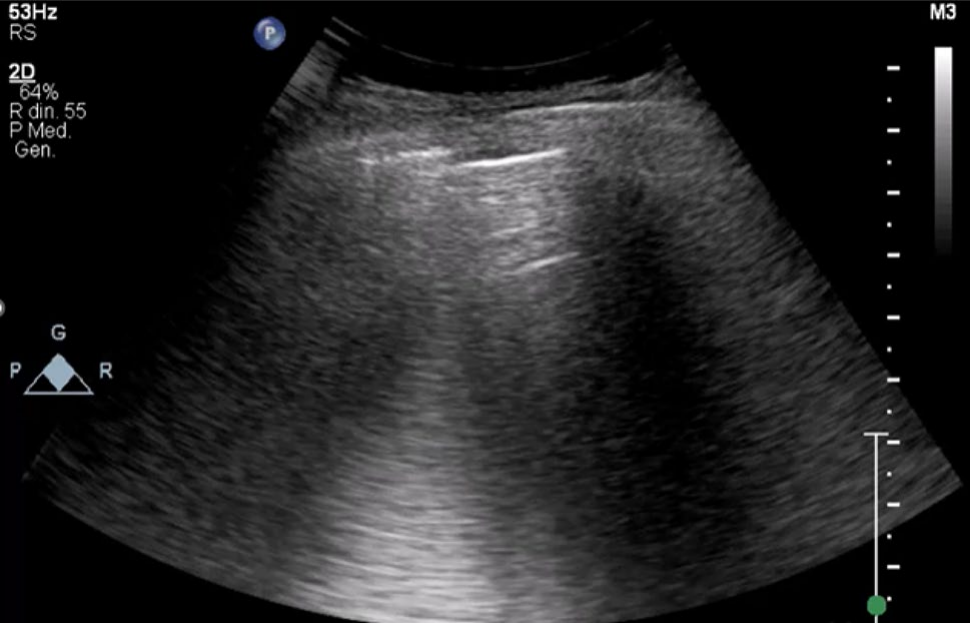
Image of pneumoperitoneum: air artifacts (similar to lung artifacts) are seen beneath the diaphragm in an intraperitoneal location. The patient is examined in the left lateral decubitus position with a right coronal scan. The air artifacts obscure visualization of the liver

The main and most important sign of gastrointestinal perforation is pneumoperitoneum (free intraperitoneal gas) (Fig. 30) [92]. The “2-scan fast exam” is based on scans of the epigastrium and right hypochondrium; the systematic approach involves scans in the supine position of the epigastrium, right and left hypochondrium, umbilical area, and scans of the right hypochondrium in the left lateral position [93]. In the supine position, there are some ultrasound signs suggestive of free intraperitoneal air, including “dirty shadowing,” which refers to ring-down artifacts representing peritoneal stripe reverberations and focal air collections, and the enhanced peritoneal stripe sign, characterized by a white, highly echogenic stripe beneath the abdominal wall fascia [92]. In the left lateral position, the shifting phenomenon can be observed, which involves the movement of free intraperitoneal air, suggestive of pneumoperitoneum [94]. This phenomenon can also be elicited by applying and then releasing light pressure with the caudal part of the probe, known as the “scissors maneuver” [95]. In this way, the free gas in the epigastrium obscures the left liver lobe and adjacent abdominal structures, causing the liver to appear and disappear [95]. Additional indirect signs of perforation are: bowel wall thickening, bowel dilatation, free fluid (with fibrinoid septa) and changes in the mesenteric fat [92]. To our knowledge, no original studies have evaluated the use of POCUS for ischemic bowel diseases.

## Discussion

Across GI etiologies of abdominal pain, POCUS emerges as a radiation-sparing, rapidly deployable first-line test that can both shorten time to decision and, in selected scenarios, approach the diagnostic performance of cross-sectional imaging [40]. In appendicitis, contemporary pediatric meta-analytic data show US, CT and MRI all achieving high accuracy, with CT/MRI at the top end (sens/spec ≈0.96/0.98) and conventional US close behind (0.93/0.89); POCUS is somewhat less sensitive but highly specific in pooled pediatric analyses and can add value for rapid severity assessment using Puylaert staging in the ED [25–27]. These findings support an ultrasound-first approach in children and adolescents, reserving CT or MRI for equivocal scans or complications.

For diverticulitis, the newest systematic review confirms high overall US performance (sens 92.5%, spec 87.7%; LR + ≈8, LR − ≈0.08), with POCUS demonstrating higher sensitivity than radiology-performed US in subgroup analyses [33]. Primary studies refine these signals: accuracy varies by disease location (notably lower in cecal disease) and declines with higher BMI, while targeted surgeon- or ED-performed POCUS can stage Hinchey *I*–II with good concordance to contrast-enhanced CT [34–36]. These nuances argue for protocolized scanning (compressibility, mural thickness/stratification, pericolic fat, Doppler hyperemia, complications) and a low threshold for CT when anatomy or habitus limit sonographic windows.

Operational benefits are most explicit in SBO. A recent ED cohort associated POCUS use with markedly shorter time-to-diagnosis and reduced ED length of stay and radiography use, without increasing hospital length of stay [64]. From a test-characteristics standpoint, IPD meta-analysis shows high sensitivity (≈83%) and specificity (≈93%), but performance is operator- and patient-dependent: attendings outperform residents, and BMI ≥ 30 kg/m^2^ attenuates sensitivity [65]. Multicenter data likewise show strong sensitivity at the bedside (≈0.88) with specificity improving substantially when cine loops are reviewed by experts (≈0.82), and they highlight the relative value of specific sonographic signs—dilated small bowel ≥ 25 mm and abnormal peristalsis (more sensitive) versus transition point, free fluid, and wall edema (more specific) [66]. Together, these data support POCUS as an ED accelerator for suspected SBO, with CT reserved for equivocal studies, suspected strangulation/ischemia, or pre-operative mapping.

In children with suspected intussusception, POCUS performance is consistently excellent: pooled sensitivity and specificity ≈95–98%, with LR + values typically > 40–100 and LR − ≈0.03–0.05; multicenter paired testing suggests POCUS accuracy can be statistically non-inferior to radiology-performed ultrasound when undertaken by experienced sonologists [67–70]. Single-center work shows high concordance even among novice pediatric emergency physicians, and ED cohorts illustrate how POCUS helps distinguish small bowel–small bowel (often transient) from ileocolic intussusception, aligning imaging with urgency of intervention [71, 72]. These findings endorse POCUS as a near-definitive front-door test in pediatric pathways, with radiology involvement for reduction or complex cases.

IBD represents another domain where bedside bowel ultrasound is clinically meaningful. Gastroenterologist-performed BUS demonstrates high sensitivity for bowel wall thickening and Crohn-related complications (stenosis, inflammatory mass), and consensus statements emphasize standardized measures (wall thickness, vascularity, mesenteric fat, complications) to track activity and complications [47, 56, 57]. Given its repeatability and lack of radiation, ultrasound can complement biomarkers and endoscopy while curbing CT use, particularly during flares or when monitoring response.

Two additional themes recur. First, sonographic recognition of free intraperitoneal air and mesenteric ischemia, while operator-dependent, has characteristic patterns (reverberation/comet-tail under the diaphragm, absent/reduced mural perfusion, bowel wall thickening, pneumatosis, portal venous gas) that can expedite escalation of care when correlated with clinical instability [59]. Second, evidence remains uneven: despite practical know-how for dynamic assessment of groin hernias, we found no original diagnostic-accuracy studies of POCUS specifically for inguinal or femoral hernias, and similarly sparse data for epiploic appendagitis. (Published reports largely comprise reviews, technical notes, case series, or studies focused on ultrasound-assisted reduction rather than diagnostic accuracy). These gaps merit prospective ED-based accuracy and workflow studies.

**Fig. 31:**
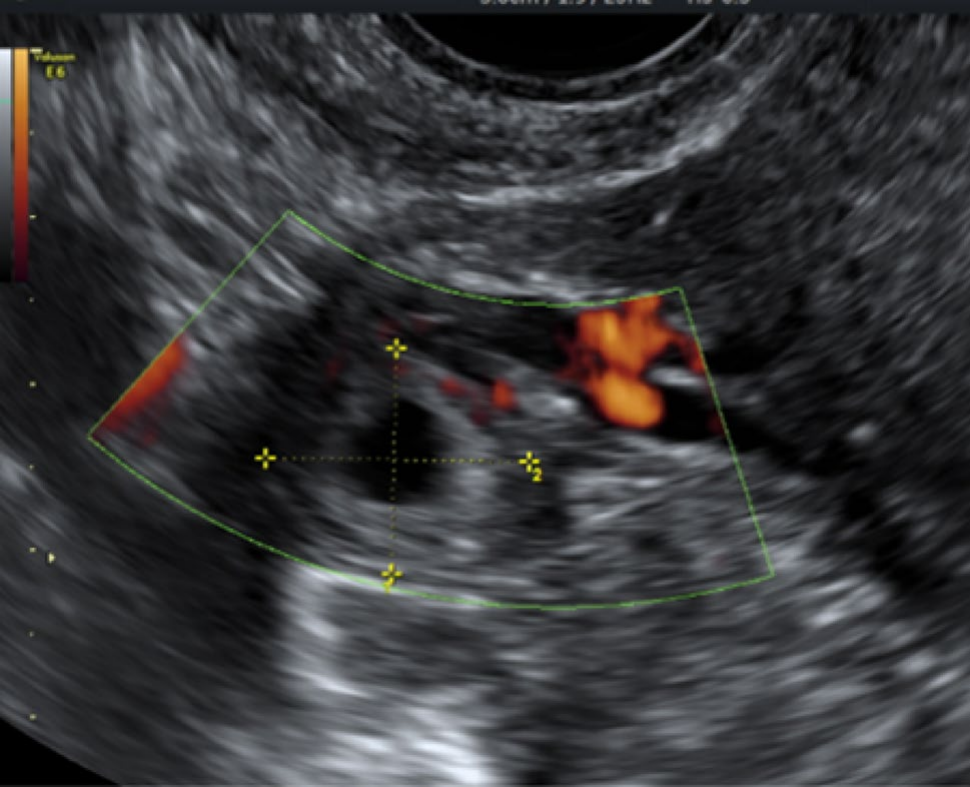
Transverse color Doppler scan showing a hyperechoic tubal ring of an ectopic gestation with no blood flow in the echogenic ring surrounding the gestational sac

**Fig. 32:**
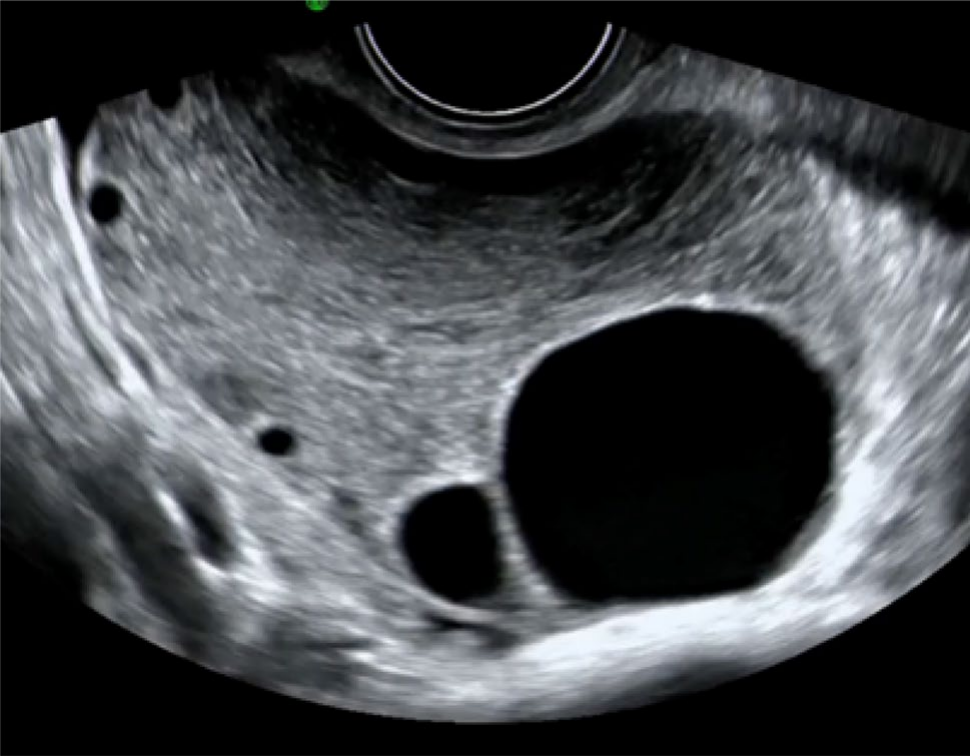
Ovarian torsion showing an enlarged and edematous with heterogeneous echotexture parenchyma with peripherally located follicles (“string of pearls” sign) and perifollicular hyperechoic rim (“follicular ring sign”)

While this pictorial review is primarily focused on gastrointestinal causes of acute pelvic pain, it is important to recognize that gynecological emergencies often present with similar symptoms and should be part of the differential diagnosis in the emergency setting. Ultrasound plays a pivotal role in this context. Ectopic pregnancy (Fig. 31), the most time-critical diagnosis, can be suggested by the presence of an adnexal mass, tubal ring sign, or free fluid in the pouch of Douglas in a patient with positive β-hCG. Ovarian torsion typically manifests as an enlarged ovary (> 4 cm) with peripheralized follicles, stromal edema, and reduced or absent vascular flow on color Doppler (Fig. 32). A ruptured ovarian cyst may appear as a collapsed cyst wall with surrounding free fluid or hemoperitoneum. Including these entities highlights the versatility of ultrasound in rapidly distinguishing gynecological from gastrointestinal causes of acute pelvic pain, ensuring timely management and improved patient outcomes.

Limitations of the current literature include heterogeneity in operator training and protocols, mixed or delayed reference standards, and spectrum bias—each of which can inflate accuracy or obscure fail points. Accordingly, ultrasound should be embedded in explicit clinical pathways that (i) standardize views and measurements; (ii) define clear “positive,” “negative,” and “indeterminate” criteria; (iii) mandate CT/MRI when red flags (ischemia, perforation) or indeterminate scans persist. Where adopted, such pathways are likely to sustain the dual advantages of POCUS—speed and safety—without compromising diagnostic certainty [96, 97]. Finally, the performance and safety of POCUS are operator-dependent [98]. We explicitly endorse formal training, documented competency, and adherence to established curricula and practice standards to ensure high-quality examinations and appropriate integration with radiology-performed ultrasound and cross-sectional imaging [97, 99].

## Conclusions

In conclusion, ultrasound plays a critical role in the differential diagnosis of acute abdominal pain by providing a rapid, non-invasive, and cost-effective imaging modality. In addition to acute diagnosis, ultrasound plays a fundamental role in monitoring the evolution of these pathologies.

## Data Availability

Data are available upon request from authors.
